# Tai chi improves balance performance in healthy older adults: a systematic review and meta-analysis

**DOI:** 10.3389/fpubh.2024.1443168

**Published:** 2024-11-11

**Authors:** Zhibo Cui, Jin Xiong, Zhihua Li, Chengbo Yang

**Affiliations:** School of Sport and Training, Chengdu Sport University, Chengdu, China

**Keywords:** tai chi, balance performance, healthy older, aging adults, meta-analysis

## Abstract

**Background:**

Previous research has indicated that tai chi exercise can effectively enhance balance performance in patients; however, its impact on healthy individuals remains uncertain. Therefore, this meta-analysis aims to investigate the effects of different intensities and styles of tai chi exercise on the balance performance of healthy older adult individuals.

**Methods:**

A targeted search method was employed to identify studies investigating the impact of tai chi exercise on balance in older adults across a range of databases, including Web of Science, PubMed, Cochrane Central, EBSCO, CHKI, and Embase. The studies were conducted in accordance with the PRISMA and PERSiST guidelines, and two independent reviewers were responsible for the search, screening of results, extraction of data, and assessment of study quality. A random-effects model was employed to calculate the weighted mean difference (WMD) and 95% confidence interval (CI).

**Results:**

2000 participants who met inclusion criteria were included in analyses across 28 trials. The findings indicated that tai chi can enhance the balance performance of healthy older adults, as demonstrated by the Timed Up and Go test (WMD = −1.04, 95% CI: −1.36 to-0.72, *p* < 0.00001, I^2^ = 71%), functional Reach test (FR) (WMD = 2.81, 95% CI: 1.60 to 4.02, *p* < 0.00001, I^2^ = 56%), and Berg Balance Scale (WMD = 2.55, 95% CI: 1.19 to 3.91, *p* = 0.0002, I^2^ = 88%), as well as other balance tests such as SLS (WMD = 5.03, 95% CI: 3.08 to 6.97, *p* < 0.00001, I^2^ = 85), and GS (WMD = 0.09, 95% CI: 0.05 to 0.12, *p* < 0.00001, I^2^ = 54%). Subgroup analyses showed that tai chi exercise for both ≤12 and > 12 weeks was statistically significant (< 0.01, respectively) for balance performance in healthy older adults, especially for tai chi exercise performed more than twice a week (WMD = −1.03, 95% CI: −1.35 to −0.72, *p* < 0.00001) and for more than 45 min each time (WMD = −1.11, 95% CI: −1.58 to-0.63, *p* < 0.00001) tai chi exercise had greater benefits on TUG time, FR distance and BBS in healthy older adults. In addition, compared to Sun-style tai chi, Yang-style tai chi was more effective.

**Conclusion:**

The tai chi exercise positively affects the balance performances of healthy older adults. Engaging in short-term (≤12 weeks) exercise for more than two 45-min sessions per week has been found to produce more pronounced effects. The effectiveness of Yang-style tai chi is superior to that of Sun-style tai chi.

**Systematic review registration:**

PROSPERO ID is CRD42024532577 https://www.crd.york.ac.uk/prospero/.

## 1 Introduction

As the aging population increases, the significance of preserving balance in older adults is becoming increasingly evident within the realm of geriatric health (1). Even among older adults who are considered healthy, balance may decline with age (2). The decline in balance not only heightens the likelihood of falls but also predisposes individuals to a variety of physical injuries. The 2021 report from the World Health Organization indicates that falls are the second most common cause of unintentional injury-related fatalities among the older adult population, exhibiting a notable surge not only in fall occurrences but also in mortality rates, with the greatest impact being observed among individuals aged 60 and above (3). This issue not only presents a significant risk to the physical well-being of older adult individuals but also significantly hinders their mobility and diminishes their overall quality of life (4). Consequently, it is imperative to explore efficacious interventions aimed at enhancing balance in the healthy older adults.

In recent years, the role of physical exercise in improving the balance of the older adult has received considerable attention (5). Among these, tai chi (TC), a traditional Chinese physical activity with a long history and cultural heritage, has been widely welcomed and favored among the older adult population due to its unique respiratory regulation mechanism, soft and smooth movement design, and high emphasis on physical and mental coordination (6). Multiple studies have consistently shown that TC is effective in enhancing the balance of older adult individuals (7, 8). Specifically, TC has been found to decrease the likelihood of falls and improve static balance in older adults (9). In addition, a recent expert consensus also recommends TC as an important part of balance training (10). The practice of TC necessitates the synchronization of respiratory and motor functions, potentially activating brain regions associated with balance maintenance, thereby enhancing overall stability (11). Additionally, TC has been demonstrated to be more effective than other forms of exercise in improving balance in this population (12, 13). Tai chi exercises involve full-body rotations and trunk twists that strengthen lower limb muscles and enhance joint flexibility, aiding older adults in body support and stability (14). These findings indicate that TC is a beneficial and safe exercise modality for older adults seeking to maintain or improve their balance.

However, a comprehensive assessment of the effects of TC on balance in older adults necessitates a more profound comprehension of the definition of balance performance and the methodologies employed for its evaluation. Balance performance can be defined as an individual’s capacity to respond and adapt to a range of body movements and external challenges while maintaining a stable center of gravity (15, 16). This capacity is task-specific and involves various components, such as static balance and dynamic balance (17). Furthermore, Shumway-Cook and Woollacott suggest that the effects of TC on balance outcomes may differ depending on the assessment tools chosen (18). Therefore, employing a variety of assessment tools and methodologies is crucial for obtaining more precise and comprehensive assessment results. For instance, the one-leg stand test, the timed rise test, and the step speed test are employed to provide a comprehensive reflection of the balance performance of the older adult, thereby establishing a scientific basis for the effect of TC in improving balance (19, 20).

In recent four review studies have been conducted to assess the efficacy of TC in improving balance in older adults. The studies revealed that TC significantly reduces the frequency of falls in older adults and effectively reduces anxiety about falls in sarcopenia and frail older adults (21, 22). Moreover, these studies highlighted that TC demonstrated clearer effects in reducing fall rates and improving Berg Balance Scale scores compared to other physical activities (23, 24). Although existing research has demonstrated the positive effects of TC on balance in older adults, the majority of studies have focused on specific subgroups with health problems, such as sarcopenia or frailty. There is a dearth of studies involving healthy older adults and a paucity of meta-analyses designed to assess the differential effects of TC on various aspects of balance. The enhancement of balance is a universal concern for individuals irrespective of their medical status. Therefore, this study aims to assess the Weighted Mean Difference (WMD) of TC on balance in healthy older adults by using META analysis and synthesizing the evidence from the included studies and to explore the quantitative effects of TC style, exercise frequency, and duration on these parameters. This will provide a scientific basis for the development of a personalized exercise intervention program to further improve the well-being and quality of life of healthy older adults.

## 2 Methods

The systematic review and meta-analysis were conducted in strict adherence to PRISMA statement and the PERSiST guidance (25, 26). The PRISMA checklist can be accessed in the Supplementary Tables S9, S10. Furthermore, the entire review process was formally documented and registered in the PROSPERO database, under the unique registration number (CRD42024532577).

### 2.1 Search strategy

A search of PubMed (*n* = 511), Web of Science (*n* = 1,036), Cochrane Central (*n* = 572), EBSCOhost (*n* = 1,553), CHKI (*n* = 190), and Embase (*n* = 654) databases covering the period from inception to January 22, 2024, was performed published literature. The search strategy was a Boolean logic search with the following search strategies: (“Tai Ji” OR “Tai-ji” OR “Tai Chi” OR “Tai Ji Quan” OR “Taijiquan” OR “T’ai Chi”) AND (Falls OR Falling OR “Fall Risk” OR Balance OR Gait OR Walking) AND (Aged OR Aging OR older adults OR older adult OR “older people”). Literature was searched without restriction on language and type of literature. In addition, citations for each included study were reviewed to ensure that no relevant articles were missed during the search. The full search strategies are provided in the Supplementary Tables S1–S6.

### 2.2 Eligibility criteria

The search criteria developed in accordance with the PICO(S) strategy are as follows (27):

1. Participants: healthy older adults with a mean age ≥ 60 (28).
2. Intervention: any form of tai chi exercise.
3. Comparison: the baseline condition of the subjects before the experiment.
4. Outcome: balance performance (an ability to maintain the body’s center of gravity) (29). The outcome included the single leg stance, timed up and go test, gait speed, functional reach test, and Berg balance scale. Among these, the Timed Up and Go (TUG) test is a widely utilized assessment tool for evaluating dynamic balance in older adults (30). The test comprises a circuit in which the subject must rise from a chair, walk a distance of three meters, turn around, and walk back to the chair to resume their seated position (31).

### 2.3 Exclusion criteria

Excluded studies included those without TC exercise intervention, participants under 60 years old, diseased populations, incomplete or missing data, lack of relevant data, duplicate studies, reviews, observational studies, case studies, qualitative studies, and non-eligible publication types like editorials, letters to the editor, erratum, study protocols, and preprint papers. The literature search did not apply any filter or exclusion criteria based on gender.

### 2.4 Selection process and data extraction

We followed PRISMA statement guidelines when extracting data and selecting studies (32). The articles from each source were integrated into a unified database using EndNote 20 software (Clarivate, The EndNote Team, Pennsylvania, United States), and duplicates were eliminated. Two reviewers (ZC and JX) independently screened the titles and abstracts retrieved from the search strategy. Any disagreement regarding the inclusion or exclusion of studies was further assessed by a third reviewer (ZL). Each author (ZC, JX, ZL, CY) conducted an individual assessment of the full-text articles according to the eligibility criteria and performed the data extraction. When the data could not be extracted, we reached out to the article author to address the issue. Otherwise, the platform was utilized to extract data from WebPlotDigitizer (33). Furthermore, we will offer comprehensive training for new members of the review team who lack familiarity with the software and content areas utilized (34).

### 2.5 Assessment of study quality

The researchers, ZC and JX, each performed an independent quality assessment of the included studies using the Cochrane Risk of Bias Tool (35). During this process, consensus was sought through in-depth discussion of any disagreements that arose from the assessment. If there were differences that could not be resolved through discussion, a third reviewer (CY) was consulted to ensure the objectivity and accuracy of the results. The Cochrane Risk of Bias tool assesses bias, which includes selection bias, performance bias, detection bias, attrition bias, reporting bias, and other bias. Each of these domains was rated as low risk, unclear risk, or high risk (36).

### 2.6 Data synthesis and analysis

Data synthesis and analysis were conducted with the use of Review Manager 5.4 (Cochrane Collaboration in Oxford, UK) and Stata SE V.15 software (Stata Corp). Random effect models were utilized to calculate the weighted mean difference (WMD) and the 95% confidence interval (CI) of TC exercise based on the Mean ± SD in each balance performance outcome (37). WMD can be negative or positive depending on the respective outcome measure. The variability in research studies was evaluated by utilizing the I^2^ statistic and categorized as insignificant (0–40%), moderate (30–60%), significant (50–90%), and substantial (75–100%) (38). We thoroughly evaluated the relevant *p*-values, particularly when heterogeneity was observed in the overlapping intervals (38, 39). If the heterogeneity test yielded a non-significant result, a fixed-effects model utilizing the Mantel–Haenszel method was employed (25). Conversely, in instances of significant statistical heterogeneity (I^2^ ≥ 50% or *p* < 0.1), a random-effects model was adopted (40, 41). Furthermore, in cases of substantial heterogeneity (I^2^ > 50%), subgroup analyses and sensitivity analyses were conducted to further elucidate the findings (42). In the subgroup analysis, we investigated the effects of TC on styles (sun and yang), exercise period ≤12 weeks and > 12 weeks weekly frequencies (≤2 days/Week and > 2 days/Week, and duration ≤45 min/session and > 45 min/session) (43). The leave-one-out method of sensitivity analysis entails the removal of each study from the analyses one by one (44). Finally, the potential for publication bias was evaluated through the use of funnel plots and Egger’s test (45).

## 3 Results

### 3.1 Study selection

**Figure 1:**
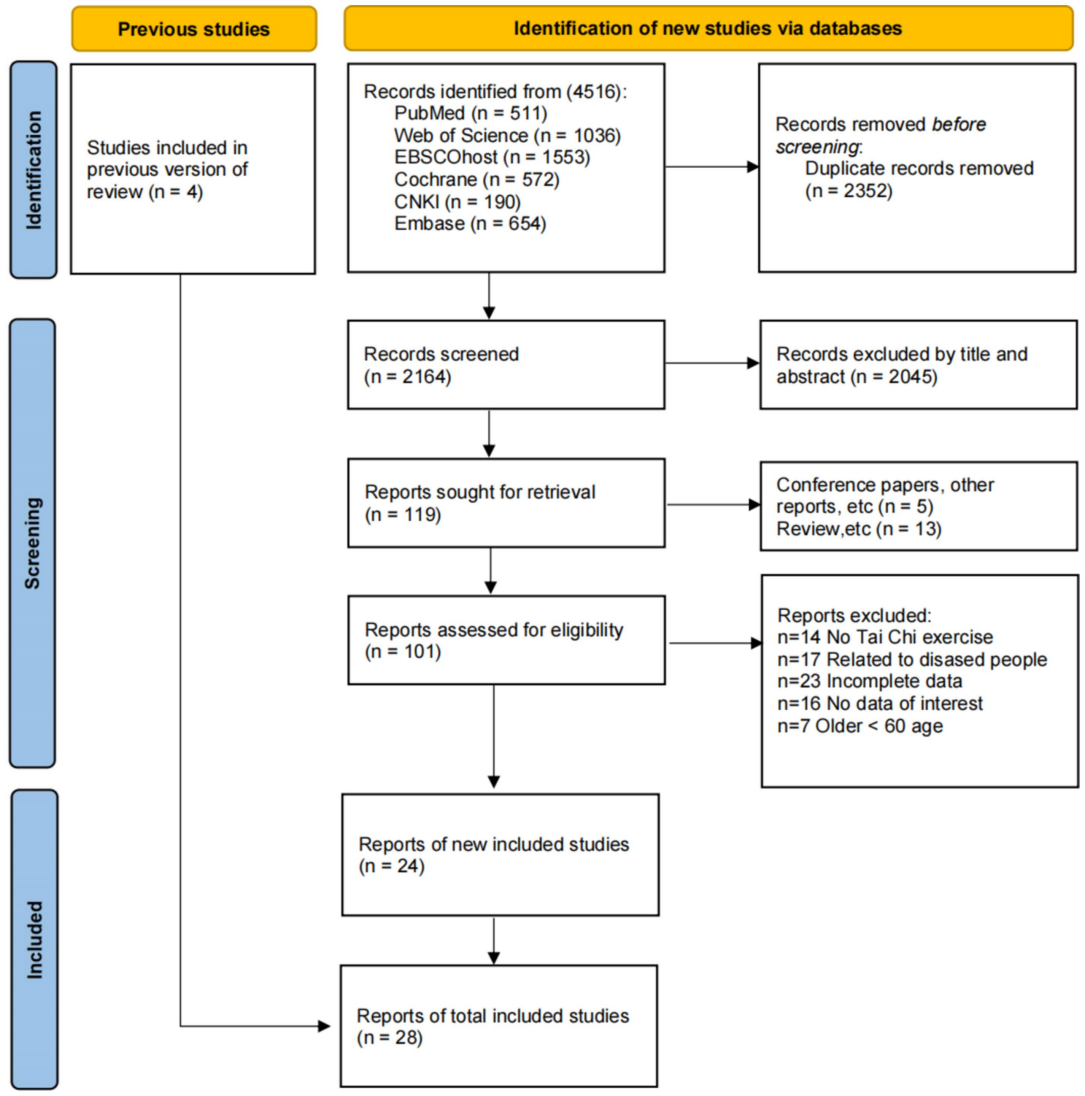
Study selection flowchart in PRISMA.

As depicted in Figure 1, a total of 4,516 articles were initially searched; as depicted in Figure 1, a total of 4,516 articles were initially searched, from which 2,352 duplicates were eliminated. Subsequent screening of titles and abstracts led to the exclusion of an additional 2045 articles. Among the remaining 119 articles, 18 were identified as conference papers, reports, or reviews, 14 articles not focused on TC exercise, 17 articles involving diseased populations, 7 articles with participants under 60, and 39 articles with incomplete or irrelevant data. In addition, 4 other relevant studies were included from references of previous reviews. Ultimately, 28 articles were included for analysis, with one or more outcomes (11, 46–72).

### 3.2 Characteristics of the included studies

There is a summary of the main characteristics and results of each of the studies that were included in Table 1. The sample sizes in the 28 articles published from 2005 to 2023 varied from 11 to 453 individuals. 2000 participants were involved in the research, with an average age of 68.24 years. These studies were conducted in diverse geographic regions, including Asia, North America, and Europe, and one study from New Zealand in Oceania. The primary styles of TC examined in the literature were Sun-style and Yang style, with one study utilizing the Chen style and the remaining studies not specifying a particular style. The period of interventions in the studies ranged from 8 to 16 weeks, including 6 studies that extended beyond five months. Exercise frequencies varied from 1 to 4 times per week, with session duration ranging from 30 to 90 min. Outcome measures included time up in 17 studies (11, 46, 49, 51, 52, 57, 59–64, 66, 69–72) berg balance scale in 9 studies (11, 51, 54–56, 58, 66, 67, 70), functional reach test in 11 studies (11, 46, 49, 50, 59–65), 11 studies (50, 51, 59, 61–64, 66, 69, 70, 72) reported single leg stance and gait speed in 7 studies (47, 48, 50, 53, 56, 64, 68).

**Table 1:** Characteristics of studies included in the meta-analysis.

Study ID	Country	Total	Age	Type	Exercise period	Frequency	Duration	Mains results
Bubela et al. (46)	American	13	71.2 ± 6.1	TC	16 weeks	3 days/week	60 min/session	①③
Chen et al. (11)	China	14	72.2 ± 2.8	Yang-style TC	8 weeks	3 days/week	30 min/session	①②③
		14	75.1 ± 5.5	Yang-style TC				
Chen et al. (47)	China	12	67.7 ± 0.7	Yang-style TC	16 weeks	4 days/week	60 min/session	④
Ge et al. (48)	China	32	70.2 ± 5.4	Yang-style TC	8 weeks	4 days/week	60 min/session	④
Huang et al. (49)	China	32	71.4 ± 0.5	TC	>5 months	1 day/week	40 min/session	①③
Kim et al. (50)	Korea	23	71.4 ± 3.3	Sun-style TC	12 weeks	2 days/week	60 min/session	③④⑤
Li et al. (51)	American	115	76.9 ± 4.7	Yang-style TC	6 months	3 days/week	40 min/session	①②⑤
Li et al. (52)	China	224	77.5 ± 5.6	TC	24 weeks	2 days/week	60 min/session	①
Liye et al. (53)	China	32	67.9 ± 3.2	Yang-style TC	8 weeks	3 days/week	90 min/session	④
Logghe et al. (54)	Netherlands	138	77.5 ± 4.7	Yang-style TC	13 weeks	2 days/week	60 min/session	②
Logghe et al. (55)	Netherlands	138	76.9 ± 4.5	Yang-style TC	13 weeks	2 days/week	60 min/session	②
Manor et al. (56)	American	29	81.0 ± 5.0	Yang-style TC	12 weeks	2 days/week	60 min/session	②④
Marcia et al. (57)	American	199	75.3 ± 8.2	TC	12 weeks	3 days/week	60 min/session	①
Mortazavi et al. (58)	Iran	27	67.2 ± 5.4	Yang-style TC	10 weeks	3 days/week	60 min/session	②
Ni et al. (59)	American	11	70.3 ± 5.7	Chen-style TC	12 weeks	2 days/week	60 min/session	①③⑤
Penn et al. (60)	China	20	76.5 ± 8.7	Yang-style TC	8 weeks	3 days/week	30 min/session	①②③
		15	75.3 ± 5.2	Yang-style TC	8 weeks	3 days/week	30 min/session	①③
Pluchino et al. (61)	American	14	69.28 ± 6.03	Sun-style TC	8 weeks	2 day/week	60 min/session	①③⑤
Roberson et al. (62)	Czech Republic	15	76.8 ± 9.4	TC	10 weeks	2 days/week	60 min/session	①③⑤
Sadeghian et al. (63)	Iran	18	65.3 ± 3.6	Sun-style TC	8 weeks	3 days/week	60 min/session	①③⑤
Son et al. (64)	Korea	21	72.8 ± 4.7	Sun-style TC	12 weeks	2 days/week	60 min/session	①③④⑤
Takeshima et al. (65)	Japan	25	72.0 ± 5.0	Yang-style TC	12 weeks	2 days/week	60 min/session	③
Taylor et al. (66)	New Zealand	233	75.3 ± 7.0	Sun-style TC	20 weeks	1 day/week	60 min/session	①⑤
		220	74.4 ± 6.2	Sun-style TC	20 weeks	2 days/week	60 min/session	
Tousignant et al. (67)	Canada	76	79.1 ± 6.4	TC	15 weeks	2 days/week	60 min/session	②
Wayne et al. (68)	American	87	64.5 ± 7.4	Yang-style TC	6 months	2 days/week	30 min/session	④
Wu et al. (68)	American	22	76.1 ± 7.9	Yang-style TC	15 weeks	4 days/week	45 min/session	①⑤
Yildirim et al. (70)	Turkey	30	62.9 ± 6.5	Yang-style TC	12 weeks	3 days/week	60 min/session	①②⑤
Zhao et al. (71)	China	20	70.2 ± 3.9	Yang-style TC	16 weeks	3 days/week	90 min/session	①
Zhu et al. (72)	China	16	67.4 ± 4.0	TC	16 weeks	4 days/week	60 min/session	①⑤

TC: Tai Chi; ① TUG: Timed Up and Go test (s); ② BBS: Berg Balance Scale (range, 0–56); ③ FR: Functional Reach (cm); ④ GS: gait speed (m/s); ⑤ SLS: Single Leg Stance (s).

### 3.3 Risk of bias

**Figure 2:**
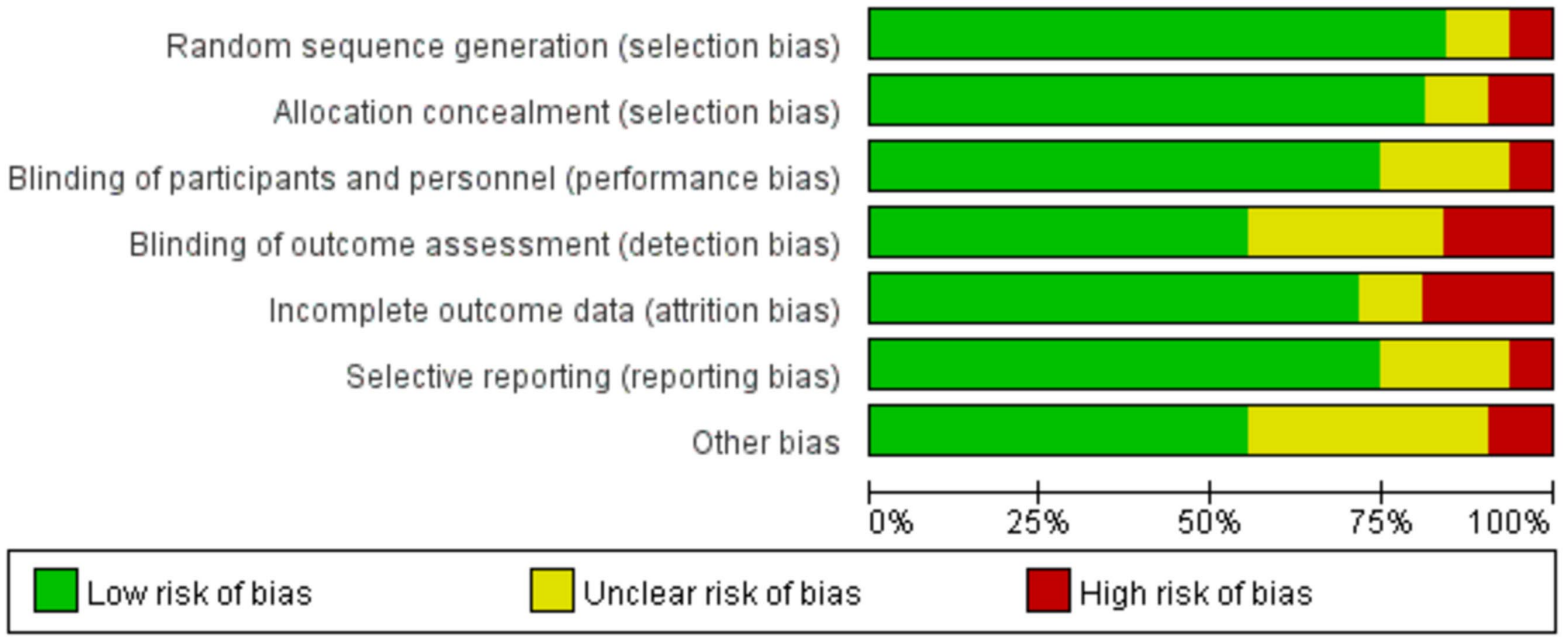
Risk of bias graph.

The Cochrane tool for risk assessment was utilized to assess the methodological quality of the literature included. For random sequence generation and allocation concealment, 23 (11, 47–56, 58–61, 63–69, 71, 72) and 22 (11, 47–53, 55, 56, 58, 59, 61, 63–72) articles were classified as low risk, respectively. Regarding performance bias, 20 articles (11, 47–49, 51–56, 59–62, 64–66, 69, 71, 72) were rated as low risk, while 6 (46, 50, 58, 63, 67, 68) were unclear, and 2 (57, 70) were high risk. For detection bias, 16 articles (11, 47, 49, 50, 55, 56, 59, 61–63, 67–72) were considered low risk, 9 (46, 48, 51, 53, 54, 57, 58, 64, 65) were unclear, and 3 (52, 60, 66) were high risk. Regarding attrition bias and reporting bias, 21 (46, 48–55, 57–65, 67, 70, 72) and 22 (46, 48–52, 54–60, 63, 64, 66–72) articles were deemed low risk, respectively. Lastly, regarding the other bias, 16 articles (47, 50, 52–54, 56, 57, 60, 62–65, 67, 68, 70, 72) were low risk, 10 (46, 48, 49, 51, 55, 59, 61, 66, 69, 71) were unclear risk, and 2 (11, 58) were high risk (Figure 2; Supplementary Figure S1).

### 3.4 Publication bias

**Figure 3:**
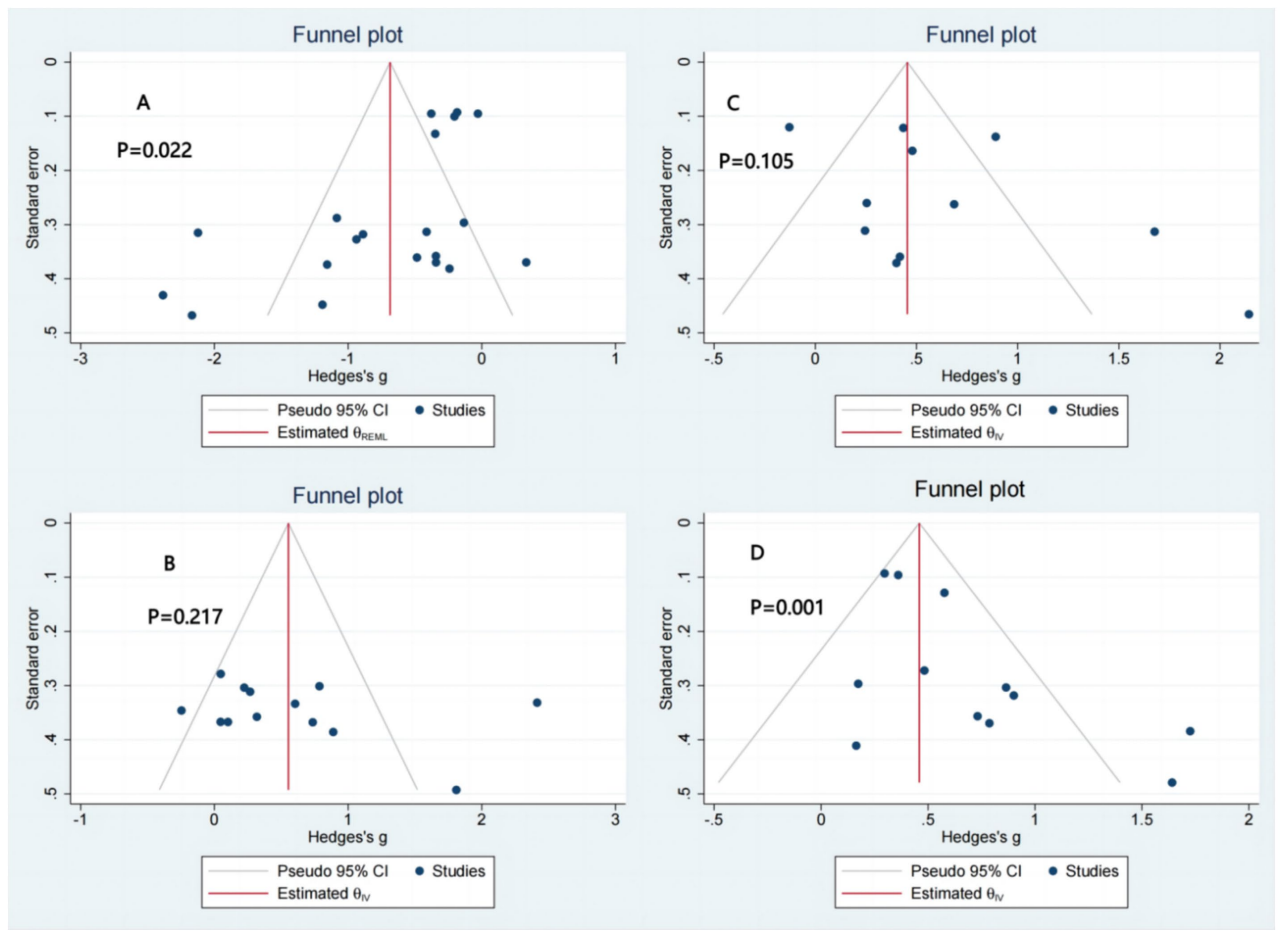
Publication bias. (A) TUG: timed up and go test. (B) FR: functional reach CI (C) BBS: berg balance scale. (D) SLS: single leg stance.

Trials comprising 10 or more items were assessed using funnel plots and Egger’s test (73). The findings indicated the absence of publication bias for BBS (*p* = 0.105) and FR (*p* = 0.217) while demonstrating publication bias for TUG (*p* = 0.022) and SLS (*p* = 0.001) (Figure 3).

### 3.5 Timed up and go test

**Figure 4:**
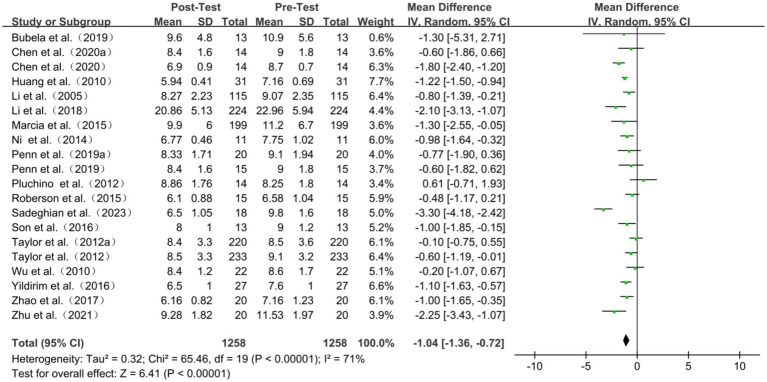
Timed up and go test of forest plot.

A total of twenty studies have reported TUG results. The results of a meta-analysis indicate that TC exercise is associated with improved TUG times in older adults. Heterogeneity was present among the studies (I^2^ = 71%, *P*<0.00001), so a random-effects model was used for analysis. The results indicated a positive effect of TC exercise on TUG time (WMD = −1.04, 95%CI: −1.36 to 0.72, *P*<0.00001) (Figure 4). After sensitivity analysis, no significant difference was found after sequentially excluding each study or studies with smaller sample sizes. Subgroup analyses showed statistically significant effects regardless of whether the exercise period was ≤12 weeks or > 12 weeks (<0.001 and *p* < 0.01, respectively). Specifically, the exercise period ≤12 weeks showed a more apparent effect on TUG time (WMD = −1.09). Furthermore, as exercise frequency increased, TC was more effective in reducing TUG time (WMD = -0.83 and-1.03, respectively). Lasting >45 min/session was more effective than those lasting ≤45 min/session (*p* < 0.001 and *p* = 0.09, respectively). Yang-style TC was more effective than Sun-style TC (*p* < 0.001 and *p* = 0.36, respectively) (Table 2; Figure 4).

**Table 2:** Timed up and go test of subgroup analyses results.

Subgroup	Included studies	WMD	95%CI	I^2^	*P*
Exercise period	17				
≤12 weeks	9	−1.09	[−1.86, −0.57]	75%	<0.001
>12 weeks	8	−1.01	[−1.41, −0.60]	60%	<0.01
Frequency	18				
≤2 days/week	8	−0.83	[−1.19, −0.47]	66%	<0.001
>2 days/week	9	−1.03	[−1.35, −0.72]	73%	<0.001
Duration					
≤45 min/session	5	−0.54	[−1.17, 0.09]	82%	0.09
>45 min/session	12	−1.11	[−1.58, −0.63]	77%	<0.001
Tai chi style	18				
Sun	4	−0.61	[−1.91, 0.70]	93%	0.36
Yang	7	−0.87	[−1.20, −0.53]	31%	<0.001

### 3.6 Functional reach test

**Figure 5:**
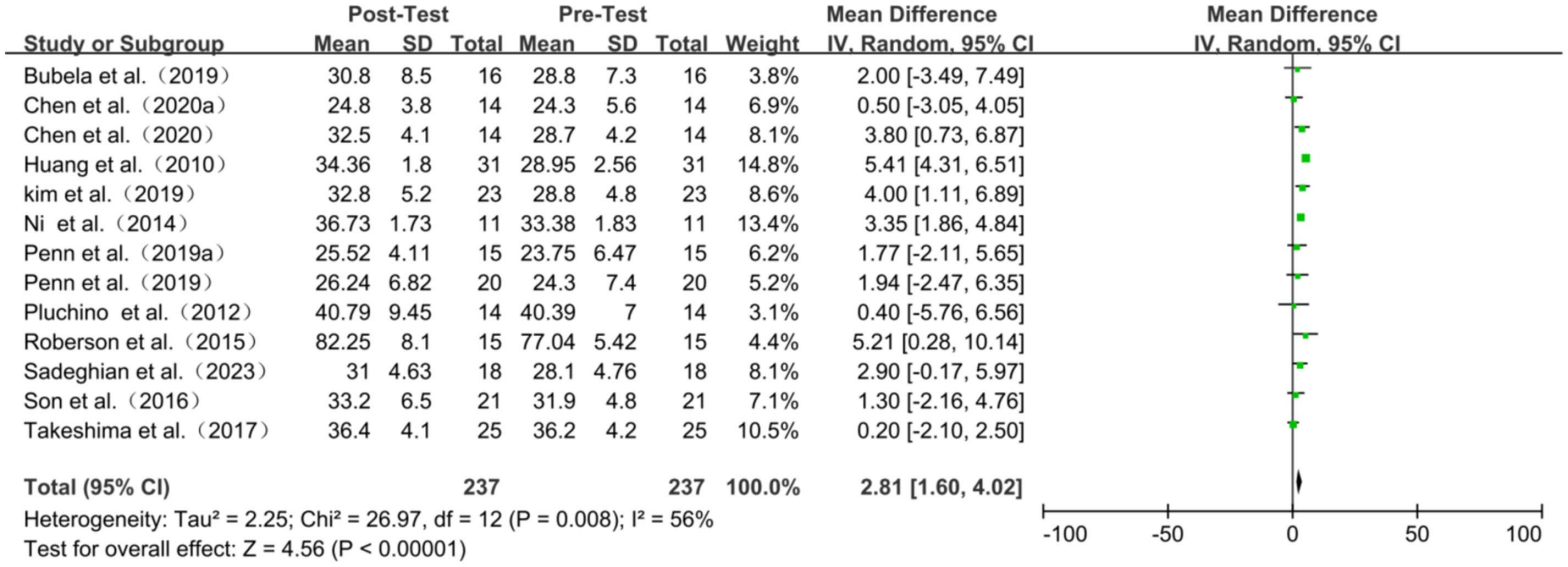
Functional reach test of forest plot.

Eleven studies encompassing thirteen comparisons investigated the impact of TC on the Functional Reach test (FR). Tests for heterogeneity indicated moderate variability among the studies (I^2^ = 56%, *p* = 0.008), necessitating the use of a random effects model for the analysis. The meta-analysis results demonstrated that TC could improve the FR distance in healthy older adult individuals (WMD = 2.81, 95% CI: 1.60 to 4.02, *p* < 0.00001) (Figure 5). By sequentially excluding smaller studies, the sensitivity analysis revealed no significant difference. Subgroup analyses showed that tai chi significantly enhances functional reach distance in older adults irrespective of the frequency, exercise period, or style of practice (Yang or Sun) (all *p* < 0.01). However, the intervention effect was notably more pronounced for ≤12 weeks than those exceeding 12 weeks (*p* < 0.001, *p* = 0.55) (Table 3; Figure 5).

**Table 3:** Functional reach functional test of subgroup analyses results.

Subgroup	Included studies	WMD	95%CI	I^2^	*P*
Exercise period					
>12 weeks	2	2.21	[−4.98, 9.41]	85%	0.55
Frequency	18				
≤2 days/week	7	2.53	[1.25,3.81]	27%	<0.001
>2 days/week	4	2.47	[0.14,4.80]	69%	0.04
Duration					
≤45 min/session	3	3.12	[0.96, 5.29]	63%	0.005
>45 min/session	8	2.27	[0.90,-3.64]	35%	0.001
Tai chi style					
Sun	3	2.89	[1.09, 4.69]	0%	0.002
Yang	8	2.47	[0.86,4.08]	69%	0.003

### 3.7 Berg balance scale

**Figure 6:**
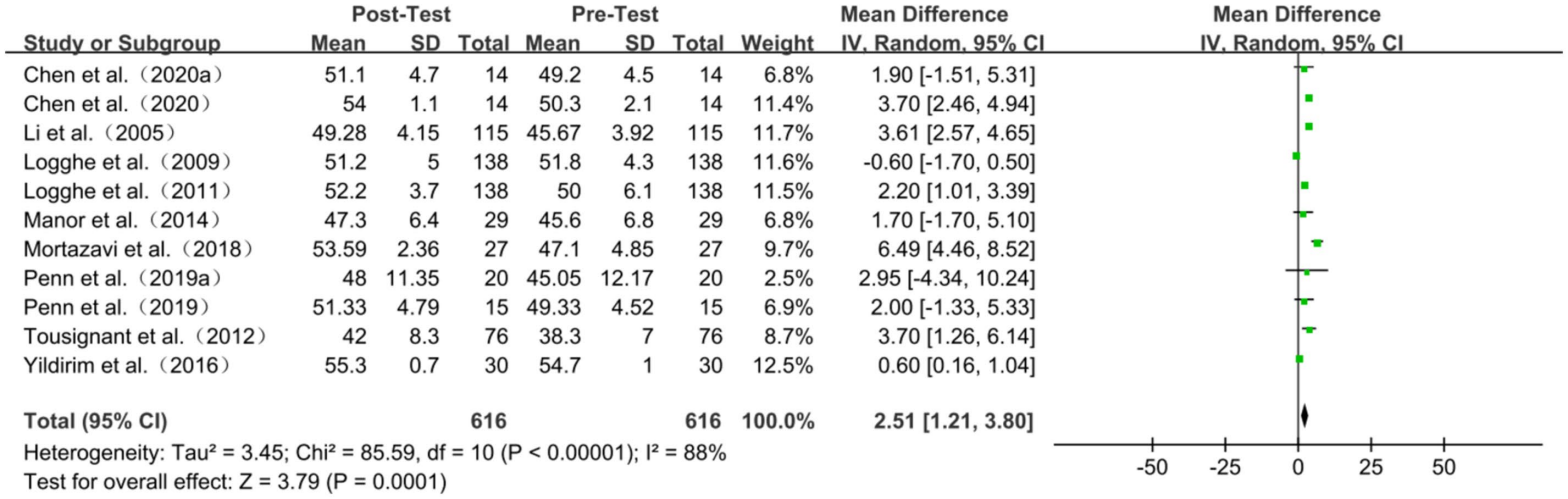
Berg balance scale of forest plot.

Nine studies reported on Berg balance scale scores. The meta-analysis results indicated considerable heterogeneity among the studies (I^2^ = 88%, *p* < 0.00001), leading to using a random effects model for effect size calculation. The findings demonstrated that TC exercise positively impacted BBS scores (WMD = 2.51, 95% CI: 1.21 to 3.80, *p* = 0.0001). The sensitivity analysis was performed sequentially, excluding studies with smaller sample sizes or those whose sample sizes were comparatively smaller. The findings of these analyses revealed that the exclusion of any given study did not yield significant variations in the overall results. Subgroup analyses showed that the performance of TC in improving BBS scores in older adults increased with the frequency of exercise (*p* = 0.12 and 0.001, respectively) (Table 4; Figure 6).

**Table 4:** Berg balance scale of subgroup analyses results.

Subgroup	Included studies	WMD	95%CI	I^2^	*P*
Exercise period					
≤12 weeks	5	3.53	[1.90, 5.16]	53%	<0.001
>12 weeks	4	2.15	[0.01, 4.28]	91%	0.002
Frequency					
≤2 days/week	4	1.60	[−0.44, 3.65]	82%	0.12
>2 days/week	5	3.07	[1.18, 4.97]	91%	0.001
Duration					
≤45 min/session	2	2.42	[0.85, 3.98]	0%	<0.001
>45 min/session	7	3.32	[2.23, 4.40]	88%	0.001

All included studies were Yang-style tai chi.

### 3.8 Other outcomes

**Figure 7:**
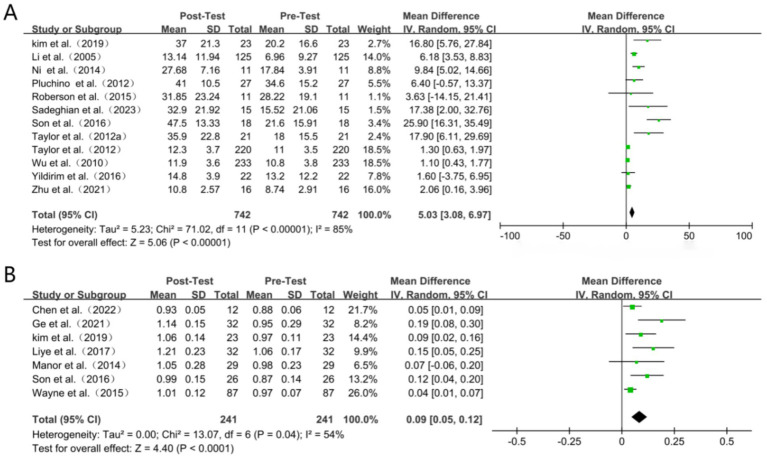
Forest plot (A) single leg stance (s). (B) Gait speed (m/s).

For other outcomes, due to limitations in the included literature, only effect sizes were combined (74). Specifically, eleven studies reported the impact of the intervention on single leg stance distance, including performance with eyes open and closed, while seven studies reported results from the gait speed test (Figure 7). The meta-analysis indicated that TC exercise led to a significant reduction in single leg stance time (WMD = 5.03, 95% CI: 3.08 to 6.97, *p* < 0.00001) and an increase in gait speed (WMD = 0.09, 95% CI: 0.05 to 0.12, *p* < 0.00001).

## 4 Discussion

The systematic review and meta-analysis, comprising 28 studies involving 2000 healthy older adults, sought to investigate the impact of TC on balance performance. The findings indicated that TC exercise has a positive effect on balance among healthy older individuals. In particular, the benefits of TC were more pronounced when the intervention period was equal to or less than 12 weeks, conducted more than two days per week, and lasted over 45 min per session. The results of the sensitivity analyses were found to be consistent with those of the primary analysis. Furthermore, Yang-style TC demonstrated superior efficacy in enhancing balance performance compared to Sun-style TC. This suggests that TC exercises may be a valuable intervention for improving balance performance in healthy older adults.

### 4.1 Effectiveness of tai chi on balance performance in healthy older adults

This study aims to elucidate the effects of TC on specific balance performance among healthy older adults. Our findings showed that TC exerts a positive influence on enhancing balance in this population. This result aligns with prior systematic reviews, which have demonstrated that TC improves balance performance and reduces fall risk in this population (75, 76). Cheng et al. performed a network meta-analysis, revealing that TC is the most efficacious among the four traditional Chinese medicine (TCM) physical therapies for fall prevention in older adults (24). Furthermore, Hu et al. (77) conducted a systematic review and meta-analysis, substantiating the positive impact of TC on neuromuscular function in the older adult population (77). However recent randomized controlled trials have demonstrated that, although TC is a neuromuscular exercise aimed at enhancing balance and coordination, it is less effective than other forms of exercise in promoting active and reactive balance strategies due to the inherently slow nature of its movements (78). These findings imply that TC may be more beneficial for improving static balance. Consequently, further research is warranted to investigate its effects on dynamic balance and the ability to respond to unexpected situations.

To elucidate the efficacy of tai chi in enhancing balance among healthy older adults, we examined the minimum clinically important difference (MCID) of the employed outcome measures. The MCID denotes the smallest threshold that signifies a clinically meaningful improvement from the patient’s perspective (79). This metric is crucial for validating the clinical significance of the treatment effect. According to the results of previous studies, the MCID for TUG was 2.1 s, FR was 8.28 cm, BBS was 3 points, SLS was 24 s, and GS was 0.13 m/s (80–84). By comparing the results of this study with the MCID criteria described above, we found that some outcomes exceeded the MCID after the tai chi intervention, while others did not (Table 5). This discrepancy may be attributed to variations in study samples. In addition, at the calculation level, prior research has predominantly established the MCID using the anchoring method, which correlates changes in the numerical scale of outcomes with an independent subjective assessment of improvement. However, due to the constraints of our study, we were limited to calculating the MCID using distribution-based methods. This method may not adequately capture clinically significant improvements that are crucial for patients and could result in varying interpretations of the effects of the same intervention (85).

**Table 5:** Studies with outcome measurements exceeded MCID.

Outcome	Reference values	Study	Total	Estimate of effect (95% CI)
TUG	2.1 s (80)	Li et al. (52)	224	−2.10 [−3.13, −1.07]
		Sadeghian et al. (63)	18	−3.30 [−4.18,-2.42]
		Zhu et al. (72)	20	−2.25 [−3.43, −1.07]
BBS	3 points (84)	Chen et al. (11)	14	3.70 [2.46, 4.94]
		Li et al. (51)	115	3.61 [2.57, 4.65]
		Mortazavi et al. (58)	27	6.49 [4.46, 8.52]
		Tousignant et al. (67)	76	3.70 [1.26, 6.14]
SLS	24 s (83)	Son et al. (64)	18	25.90 [16.31, 35.49]
GS	0.13 m/s (82)	Ge et al. (48)	32	0.19 [0.08, 0.30]
		Liye et al. (53)	32	0.15 [0.05, 0.25]

In our study, which focused on the effects of TC on balance in healthy older adults, TC improved not only static balance (e.g., SLB) but also dynamic balance (e.g., TUG and GS) and enhanced proactive balance (e.g., FR). In contrast to the observed enhancements in TUG, FR, SLS, and BBS, the impact of TC on GS appeared to be comparatively constrained. This phenomenon may be attributable to the ceiling effect, wherein the potential for improvement in GS metrics is restricted by an individual’s elevated baseline performance. Gait speed, defined as walking a certain distance on a flat surface in a short period, is typically correlated with an individual’s sports performance (86). Conversely, TC primarily comprises a sequence of slow isotonic movements, predominantly executed in a semi-deep squatting posture, and involves intricate body rotations and shifts in the center of gravity (87–89). These characteristics may diverge from the requirements of rapid, linear movement as assessed in stride speed tests, potentially influencing the efficacy of TC in improving stride speed. Furthermore, in the study of step speed, the mean step speed after TC was 1.1 m/s. Although the WMD value of 0.09 was relatively small, Montero-Odasso et al. recommend 0.7–1.0 m/s to be the mean value, with >1.1 m/s being the high value (90). Thus, TC has a small but meaningful improvement in step speed, especially in healthy older adults.

### 4.2 Most effective dose of tai chi exercise for healthy older adults

#### 4.2.1 Exercise frequency

Several studies have confirmed that physical activity and exercise effectively promote balance and prevent falls in older adults (91–93). The World guidelines for fall prevention and management for older adults recommend that older adults engage in 150–300 min of moderate-intensity physical activity or 75–150 min of vigorous-intensity physical activity each week to promote balance performance and prevent falls (5). Moreover, the World Health Organization (3) recommends that older adults engage in moderate-intensity exercise at least three days a week to promote bone health, muscle strength, and overall body function, thereby reducing the risk of falls (94). With regard to the frequency and duration of exercise, there is as yet no consensus among experts on the existing exercise guidelines for TC training for older adults. In order to address this gap, guidelines from the World Health Organization are often used as a reference in order to develop TC training programs that are suitable for older adults. A number of randomized controlled trials have demonstrated that practicing TC at least three times a week is an effective method of enhancing balance and significantly reducing the risk of falls in older adults (77, 95, 96).

This finding is further supported by our study, which showed that a variety of balance indicators in older adults improved significantly as the frequency and duration of TC practice increased. However, it is important to note that a comparable positive effect was not observed in the FR measurements. This discrepancy may be attributable to the complex interplay of multiple factors, including variability in training regimens, exercise environments, and differing levels of monitoring and recording (97). Additionally, while TC practice has demonstrated potential in improving balance, the inconsistencies observed across studies significantly complicate the establishment of a definitive relationship between the frequency of TC exercise and its intervention effects. In consideration of these findings, future research should endeavor to investigate these multifaceted factors more comprehensively, taking into account both the qualitative and quantitative aspects of exercise sessions. This approach is essential to ensure the safety and efficacy of TC interventions and to optimize their potential health benefits.

#### 4.2.2 Exercise period

The results of our study indicated that after a period of 12 weeks or less, TC training led to statistically significant improvements in balance performance (all *p* < 0.05). This result is consistent with previous studies and further validates the positive effects of TC training on balance. Yildirim et al. observed notable improvements in TUG time and Berg balance scores following a brief 8–12 week TC training program (70). Likewise, a recent systematic review indicated that TC interventions of shorter duration (<20 weeks) yielded more substantial advantages in functional flexibility and balance for healthy older individuals compared to longer-term interventions (>20 weeks) (98). This may be attributed to the fact that the short-term intervention prompted an initial adaptive response in the body, which facilitated the improvement of balance (99). Nevertheless, it is important to note that the most recent meta-analysis demonstrated that the impact of TC on improving physical balance and SLS in older adults became more pronounced as the duration of exercise increased (23). In addition, the Physical Activity Guidelines for Americans emphasize the health benefits of exercise irrespective of its duration (100). The guidelines from New Zealand offer the most comprehensive information on enhancing balance. According to this guideline, the optimal interventions for improving balance involve engaging in 60-min sessions three times per week, over a recommended period of 4 to 52 weeks (101). This means that TC has a positive effect on balance and flexibility in older people in both the short and long term. While short-term training may produce immediate improvements, long-term practice may produce more lasting and cumulative positive effects. Therefore, encouraging older adults to participate in TC on a consistent basis may be important for improving their quality of life and health.

#### 4.2.3 Tai chi type

The impact of various TC styles on balance in healthy older adults, as a multifaceted form of physical and mental exercise, warrants comprehensive investigation. Zou et al. report that Yang-style TC, characterized by slow, rhythmic movements, positively influences balance parameters and musculoskeletal flexibility in older adults (53). Taylor et al. conducted a study to assess the safety and feasibility of community-based Yang-style tai chi for individuals with chronic stroke (102). The findings indicated that Yang-style TC was safe, with participants exhibiting a high level of adherence and no reported falls or other adverse events. Furthermore, a study conducted by Tsai et al. demonstrated that a 20-week Sun-style TC program significantly enhanced balance control among older adults, with this improvement remaining unaffected by participants’ cognitive levels (103). These findings suggest that different styles of TC may differently affect balance in older adults due to their different movement characteristics and training intensities.

Our research found that practicing Yang-style TC significantly improves balance in healthy older adults, supporting previous studies that suggest it is more effective than Sun-style TC (104). Yang-style TC is distinguished by its direct movements, which may facilitate the enhancement of muscle memory and render it more accessible for older adults to master (105). Conversely, Sun-style TC is characterized by its elevated stance, narrow foot spacing, rapid movement switching, and numerous follow-up movements (106). Consequently, Yang-style TC appears to be more appropriate for beginners and older individuals with diminished balance. In contrast, Sun-style TC may be better suited for practitioners who possess foundational TC skills and seek a more challenging practice. Moreover, as one of the pioneering TC styles to gain international prominence, Yang-style TC has undergone continuous adaptation to diverse cultural contexts and health requirements. This adaptability has catalyzed the evolution of various teaching methodologies and practice styles. Consequently, the cross-cultural adaptation of Yang-style TC has enhanced its capacity to address the specific needs of different demographic groups, thereby augmenting its efficacy as a form of exercise.

### 4.3 Limitations

This study used six databases to improve the reliability of findings on the effects of TC on balance in healthy older adults. Following PRISMA guidelines and registering the protocol with PROSPERO increased the transparency and rigor of the study. However, there are some limitations of this study that need to be addressed in the future. For instance, balance assessments like the BBS depend on self-reported data, which can be affected by cognitive and memory biases, leading to potential inaccuracies and reporting bias. Future studies should use more objective measurement tools to reduce these biases. Future research should use more objective measurement tools to reduce biases. Moreover, the lack of detailed studies on reactive balance limits understanding of tai chi’s effects on it.

Regarding data analysis, some subgroup analyses were unable to draw reliable conclusions or were not performed due to limitations in the number and characteristics of included studies. This resulted in significant heterogeneity between subgroups. This heterogeneity masked some important effects and limited the ability to generalize the findings. Furthermore, while the MCID was used to evaluate tai chi’s impact on balance, varying study designs, sample traits, and MCID calculation methods can result in different interpretations of the same interventions. Lastly, this study could not provide insight into the dose response between training parameters. Different training frequencies, durations, intensities, and styles may affect balance in older adults differently. Further investigation is warranted to examine the relationships among these training parameters through the utilization of complex statistical methodologies to ascertain the most effective TC exercise regimen. In summary, based on the available evidence and noted limitations, recommendations to enhance future research on tai chi’s impact on homeostasis are provided in the Supplementary Table S8.

## 5 Conclusion

Our study quantitatively analyzed the effects of TC on balance performance in healthy older adults and updated the existing literature. The results indicated that TC significantly enhanced balance in healthy older adults, particularly with training sessions over 45 min, more than twice a week, and within a short-term period (≤12 weeks). Among the different styles of tai chi, Yang-style tai chi was superior to Sun-style tai chi in improving balance. However, due to limited research, we could not assess TC impact on reactive balance in older adults. These results mainly pertain to healthy individuals over 60, and further studies are required to confirm their relevance to other groups.

## Data Availability

The datasets presented in this study can be found in online repositories. The names of the repository/repositories and accession number(s) can be found in the article/Supplementary material.
