# Supplementary material for: Tai chi improves balance performance in healthy older adults: a systematic review and meta-analysis
Source: Front Public Health. 2024 Nov 11;12:1443168. doi: 10.3389/fpubh.2024.1443168 (PMC11586773; doi:10.3389/fpubh.2024.1443168)
Supplement: Supplementary file 1 [file Table_1.docx]

**SUPPLEMENTAL MATERIAL**

| **Tai Chi Improving Balance Performance in Healthy Older: A Systematic review and Meta-Analysis** |
| --- |

[Table S1 Search strategy detailed for PubMed 1](#_Toc26373)

[Table S2 Search strategy detailed for Web of Science 1](#_Toc5444)

[Table S3. Search strategy detailed for EBSCOhost 2](#_Toc7057)

[Table S4. Search strategy detailed for Cochrane Central 2](#_Toc4732)

[Table S5. Search strategy detailed for CNKI 3](#_Toc6519)

[Table S6. Search strategy detailed for Embase 3](#_Toc26043)

[Table S7. Excluded studies by reason for exclusion (N=95) 3](#_Toc12289)

[Table S8 Recommendations for future studies on the effect of Tai Chi on balance performance 12](#_Toc3051)

[Figure S1 Risk of bias assessment. 15](#_Toc12328)

[Table S9. PRISMA Abstracts checklist 16](#_Toc8945)

[Table S10. PRISMA checklist. 17](#_Toc21962)

**Table S1 Search strategy (Jan 9, 2024) detailed for PubMed**

| **ID** | **Query** | **Results** |
| --- | --- | --- |
| 1 | ((((Aged[Title/Abstract]) OR (Aging[Title/Abstract])) OR ("older adults"[Title/Abstract])) OR (elderly[Title/Abstract])) OR ("older people"[Title/Abstract]) | 1,262,419 |
| 2 | (((((Balance[Title/Abstract]) OR (Falling[Title/Abstract])) OR ("Fall Risk"[Title/Abstract])) OR (Falls[Title/Abstract])) OR (Gait[Title/Abstract])) OR (Walking[Title/Abstract]) | 488,747 |
| 3 | ((((("Tai Ji"[Title/Abstract]) OR ("Tai-ji"[Title/Abstract])) OR ("Tai Chi"[Title/Abstract])) OR ("Tai Ji Quan"[Title/Abstract])) OR ("Taijiquan"[Title/Abstract])) OR ("T'ai Chi"[Title/Abstract]) | 2,582 |
| 4 | ((((((("Tai Ji"[Title/Abstract]) OR ("Tai-ji"[Title/Abstract])) OR ("Tai Chi"[Title/Abstract])) OR ("Tai Ji Quan"[Title/Abstract])) OR ("Taijiquan"[Title/Abstract])) OR ("T'ai Chi"[Title/Abstract])) AND ((((((Falls[Title/Abstract]) OR (Falling[Title/Abstract])) OR ("Fall Risk"[Title/Abstract])) OR (Balance[Title/Abstract])) OR (Gait[Title/Abstract])) OR (Walking[Title/Abstract]))) AND (((((Aged[Title/Abstract]) OR (Aging[Title/Abstract])) OR ("older adults"[Title/Abstract])) OR (elderly[Title/Abstract])) OR ("older people"[Title/Abstract])) | 511 |

**Table S2 Search strategy (Jan 9, 2024) detailed for Web of Science**

| **Search** | **Results** |
| --- | --- |
| ALL=("Tai Ji") | 142 |
| ALL=("Tai-ji") | 142 |
| ALL=("Tai Chi") | 3724 |
| ALL=("Tai Ji Quan") | 52 |
| ALL=("Taijiquan") | 111 |
| ALL=("T'ai Chi") | 73 |
| #6 OR #5 OR #4 OR #3 OR #2 OR #1 | 3911 |
| ALL=(Falls) | 270033 |
| ALL=(Falling) | 240220 |
| ALL=(“Fall Risk”) | 5573 |
| ALL=(Balance) | 553513 |
| ALL=(Gait) | 69718 |
| ALL=(Walking) | 149648 |
| #8 OR #9 OR #10 OR #11 OR #12 OR #13 | 966630 |
| ALL=(Aged) | 2533092 |
| ALL=(Aging) | 2630949 |
| ALL=(older adults) | 289844 |
| ALL=(elderly) | 213900 |
| ALL=(older people) | 120537 |
| #15 OR #16 OR #17 OR #18 OR #19 | 2861890 |
| #20 AND #14 AND #7 | 1036 |

**Table S3. Search strategy (Jan 9, 2024) detailed for EBSCOhost**

| **ID** | **Search** | **Results** |
| --- | --- | --- |
| S1 | "Tai Ji" OR "Tai-ji" OR "Tai Chi" OR "Tai Ji Quan" OR "Taijiquan" OR "T'ai Chi" | 8,282 |
| S2 | Falls OR Falling OR Fall Risk OR Balance OR Gait OR Walking | 2,424,929 |
| S3 | Aged OR Aging OR older adults OR elderly OR "older people" | 7,788,381 |
| S4 | ( "Tai Ji" OR "Tai-ji" OR "Tai Chi" OR "Tai Ji Quan" OR "Taijiquan" OR "T'ai Chi" ) AND ( Falls OR Falling OR "Fall Risk" OR Balance OR Gait OR Walking ) AND ( Aged OR Aging OR older adults OR elderly OR "older people" ) | 1,553 |

**Table S4. Search strategy (Jan 9, 2024) detailed for Cochrane Central**

| **ID** | **Search** | **Results** |
| --- | --- | --- |
| #1 | ("Tai Ji"):ti,ab,kw OR ("Tai-ji"):ti,ab,kw OR ("Tai Chi"):ti,ab,kw OR ("Tai Ji Quan"):ti,ab,kw OR ("Taijiquan"):ti,ab,kw (Word variations have been searched) | 1878 |
| #2 | (Aged):ti,ab,kw OR (aging population):ti,ab,kw OR (older adults):ti,ab,kw OR (elderly):ti,ab,kw OR (older people):ti,ab,kw (Word variations have been searched) | 651449 |
| #3 | (Falls):ti,ab,kw OR (Falling):ti,ab,kw OR (Fall Risk):ti,ab,kw OR (accidental falls):ti,ab,kw OR (falls prevention):ti,ab,kw (Word variations have been searched) | 74131 |
| #4 | #1 AND #2 AND #3 | 582 |

**Table S5. Search strategy (Jan 9, 2024) detailed for CNKI**

| **Search** | **Results** |
| --- | --- |
| (tai chi + tai chi exercise + tai chi practice + tai chi + tai chi exercise) AND (balance + falls + fall risk + gait) AND (elderly + seniors) | 190 |

**Table S6. Search strategy (Jan 9, 2024) detailed for Embase**

| **ID** | **Search** | **Results** |
| --- | --- | --- |
| #1 | ('tai ji':ti,ab,kw OR 'tai-ji':ti,ab,kw OR 'tai chi':ti,ab,kw OR 'tai ji quan':ti,ab,kw OR 'taijiquan':ti,ab,kw OR 't`ai chi':ti,ab,kw) AND ('falls':ti,ab,kw OR 'falling':ti,ab,kw OR 'fall risk':ti,ab,kw OR 'balance':ti,ab,kw OR 'gait':ti,ab,kw OR 'walking':ti,ab,kw) AND ('aged':ti,ab,kw OR 'aging':ti,ab,kw OR 'older adults':ti,ab,kw OR 'elderly':ti,ab,kw OR 'older people':ti,ab,kw) | 654 |

**Table S7. Excluded studies by reason for exclusion (N=95)**

| **No Tai Chi exercise(n=14)** | |
| --- | --- |
| 1 | Brudnak MA, Dundero D, Van Hecke FM. Are the 'Hard' Martial Arts, Such as the Korean Martial Art, Taekwon-Do, of Benefit to Senior Citizens? *Medical Hypotheses*. (2002) 59:485-91. doi:10.1016/S0306-9877(02)00203-7 |
| 2 | Bruininks BD, Sage SK, Korak JA. Evaluation of Short-Term Multi-Component Exercise Programming on Major Variables That Directly Influence Fall Risk in Older Women: A Pilot Study. *Journal of Women & Aging*. (2022) 34:415-28. doi:10.1080/08952841.2021.1942701 |
| 3 | Chang C, Karagounis LG, Yu Y, Yin J, Donato-Capel L, Shevlyakova M, et al. Combining Nutritional Supplementation and Progressive Physical Activity Program Improves Functionality and Quality of Life in Healthy 50y+ Volunteers with Knee Joint Discomfort: A Baseline-Control Trial. *Osteoarthritis and cartilage*. (2017) 25:S293‐S4. |
| 4 | Carande-Kulis V, Stevens JA, Florence CS, Beattie BL, Arias I. A Cost-Benefit Analysis of Three Older Adult Fall Prevention Interventions. Journal of safety research. (2015) 52:65-70. doi:10.1016/j.jsr.2014.12.007 |
| 5 | Espinel Z, Fitzmaurice C, Kauffman M, Munoz O, Shultz J. Decreasing Loneliness and Social Isolation During the Covid Pandemic Using Cancer Support Services: Case Study of a Geriatric Patient with Diffuse Large B-Cell Lymphoma. *Psycho-Oncology*. (2022) 31:87. doi:10.1002/pon.5872 |
| 6 | Fogaca LZ, Portella CFS, Ghelman R, Abdala CVM, Schveitzer MC. Mind-Body Therapies from Traditional Chinese Medicine: Evidence Map. *Frontiers in Public Health*. (2021) 9. doi:10.3389/fpubh.2021.659075 |
| 7 | Garber CE, Blissmer B, Deschenes MR, Franklin BA, Lamonte MJ, Lee IM, et al. Quantity and Quality of Exercise for Developing and Maintaining Cardiorespiratory, Musculoskeletal, and Neuromotor Fitness in Apparently Healthy Adults: Guidance for Prescribing Exercise. *Medicine & Science in Sports & Exercise*. (2011) 43:1334-59. doi:10.1249/MSS.0b013e318213fefb |
| 8 | Hartescu I, Morgan K, Stevinson CD. Increased Physical Activity Improves Sleep and Mood Outcomes in Inactive People with Insomnia: A Randomized Controlled Trial. *Journal of Sleep Research*. (2015) 24:526-34. doi:10.1111/jsr.12297 |
| 9 | Jeter PE, Moonaz SH, Bittner AK, Dagnelie G. Ashtanga-Based Yoga Therapy Increases the Sensory Contribution to Postural Stability in Visually-Impaired Persons at Risk for Falls as Measured by the Wii Balance Board: A Pilot Randomized Controlled Trial. *Plos One*. (2015) 10. doi:10.1371/journal.pone.0129646 |
| 10 | Krause L, Farrow D, Pinder R, Buszard T, Kovalchik S, Reid M. Enhancing Skill Transfer in Tennis Using Representative Learning Design. *Journal of sports sciences*. (2019) 37:2560‐8. doi:10.1080/02640414.2019.1647739 |
| 11 | Li F, Wang DY, Ba XH, Liu Z, Zhang MQ. The Comparative Effects of Exercise Type on Motor Function of Patients with Parkinson's Disease: A Three-Arm Randomized Trial. *Frontiers in Human Neuroscience*. (2022) 16. doi:10.3389/fnhum.2022.1033289 |
| 12 | Shanahan J, Coman L, Ryan F, Saunders J, O'Sullivan K, Bhriain ON, et al. To Dance or Not to Dance? A Comparison of Balance, Physical Fitness and Quality of Life in Older Irish Set Dancers and Age-Matched Controls. *Public Health (Elsevier)*. (2016) 141:56-62. doi:10.1016/j.puhe.2016.07.015 |
| 13 | Winser SJ, Schubert MC, Chan AYY, Kannan P, Whitney SL. Can Pre-Screening Vestibulocerebellar Involvement Followed by Targeted Training Improve the Outcomes of Balance in Cerebellar Ataxia? *Medical hypotheses*. (2018) 117:37-41. doi:10.1016/j.mehy.2018.06.001 |
| 14 | Yokoi K, Yoshimasu K, Takemura S, Fukumoto J, Kurasawa S, Miyashita K. Short Stick Exercises for Fall Prevention among Older Adults: A Cluster Randomized Trial. *Disability and Rehabilitation*. (2015) 37:1268-76. doi:10.3109/09638288.2014.961660 |
| **Related to diseased(n=17)** | |
| 1 | Au-Yeung SS, Hui-Chan CW, Tang JC. Short-Form Tai Chi Improves Standing Balance of People with Chronic Stroke. *Neurorehabilitation and neural repair*. (2009) 23:515‐22. doi:10.1177/1545968308326425 |
| 2 | Barrado-Martín Y, Heward M, Polman R, Nyman SR. Acceptability of a Dyadic Tai Chi Intervention for Older People Living with Dementia and Their Informal Carers. *Journal of aging and physical activity*. (2019) 27:166-83. doi:10.1123/japa.2017-0267 |
| 3 | Bruininks BD, Sage SK, Korak JA. Evaluation of Short-Term Multi-Component Exercise Programming on Major Variables That Directly Influence Fall Risk in Older Women: A Pilot Study. *Journal of Women & Aging*. (2022) 34:415-28. doi:10.1080/08952841.2021.1942701 |
| 4 | Caminiti G, Volterrani M, Arisi A, Cerrito A, Massaro R, Bovone S, et al. Tai Chi Added to Endurance Training Vs Endurance Training Alone in Elderly Patients with Chronic Heart Failure: A Randomized Pilot Study. *European Journal of Cardiovascular Prevention and Rehabilitation*. (2010) 17:S87. |
| 5 | Ferrara PE, Maggi L, Foti C, Maccauro G, Ronconi G. Evaluation of Quality of Life and Static Balance in Postmenopausal Osteoporosis Women after Tai Chi Chuan Practice: An Observational Randomized Case Control Study. *Journal of Biological Regulators and Homeostatic Agents*. (2019) 33:163-9. |
| 6 | Gao S, Kaudimba KK, Cai J, Tong Y, Tian Q, Liu P, et al. A Mobile Phone App-Based Tai Chi Training in Parkinson's Disease: Protocol for a Randomized Controlled Study. *Frontiers in Neurology*. (2020) 11. doi:10.3389/fneur.2020.615861 |
| 7 | Hartman CA, Manos TM, Winter C, Hartman DM, Li B, Smith JC. Effects of T'ai Chi Training on Function and Quality of Life Indicators in Older Adults with Osteoarthritis. *Journal of the American Geriatrics Society*. (2000) 48:1553‐9. doi:10.1111/j.1532-5415.2000.tb03863.x |
| 8 | Kaplan H, Brooks R, Cassone P, Estepan H, Hore P, Knott V, et al. The Effects of Tai-Chi on Seniors with Developmental Disabilities. *Physical and Occupational Therapy in Geriatrics*. (2004) 21:41-51. doi:10.1300/J148v21n04_03 |
| 9 | Kutner NG, Barnhart H, Wolf SL, McNeely E, Xu T. Self-Report Benefits of Tai Chi Practice by Older Adults. *Journals of gerontology. Series B, Psychological sciences and social sciences*. (1997) 52:P242‐6. doi:10.1093/geronb/52b.5.p242 |
| 10 | Liu T, Chan AWK, Chair SY. Group- Plus Home-Based Tai Chi Program Improves Functional Health among Patients with Coronary Heart Disease: A Randomized Controlled Trial. *European Journal of Cardiovascular Nursing*. (2022) 21:597-611. doi:10.1093/eurjcn/zvab126 |
| 11 | Mihay LM, Boggs KM, Breck AJ, Dokken EL, NaThalang GC. The Effect of Tai Chi Inspired Exercise Compared to Strength Training: A Pilot Study of Elderly Retired Community Dwellers. *Physical and Occupational Therapy in Geriatrics*. (2006) 24:13-26. doi:10.1300/J148v24n03_02 |
| 12 | Ming-Chien C, James CR, Sawyer SF, Brismée J-M, Xu KT, Poklikuha G, et al. Effects of Tai Chi Exercise on Posturography, Gait, Physical Function and Quality of Life in Postmenopausal Women with Osteopaenia: A Randomized Clinical Study. *Clinical Rehabilitation*. (2010) 24:1080-90. |
| 13 | Murphy L, Riley D, Rodgers J, Plank S, Lehman S, Duryea B. Effects of Tai Chi on Balance, Mobility, and Strength among Older Persons Participating in an Osteoporosis Prevention and Education Program. *Explore (New York, N.Y.)*. (2005) 1:192-3. doi:10.1016/j.explore.2005.02.019 |
| 14 | Roberts BL, Marsiske M, Altmann L, Thomas L. The Effects of Tai Chi on Disability in Older Adults with Mobility Difficulty. *Journal of the American Geriatrics Society*. (2010) 58:S23. doi:10.1111/j.1532-5415.2010.02850.x |
| 15 | Solianik R, Brazaitis M, Čekanauskaitė-Krušnauskienė A. Tai Chi Effects on Balance in Older Adults: The Role of Sustained Attention and Myokines. *The Journal of sports medicine and physical fitness*. (2022) 62:1512-8. doi:10.23736/S0022-4707.21.12990-1 |
| 16 | Wong TWL. Feasibility and Preliminary Efficacy of Ai Chi Aquatic Exercise Training in Hong Kong's Older Adults with Risk of Falling: Design and Methodology of a Randomized Controlled Trial. *Contemporary clinical trials communications*. (2019) 15. doi:10.1016/j.conctc.2019.100376 |
| 17 | Zeeuwe PEM, Verhagen AP, Bierma-Zeinstra SMA, Van Rossum E, Faber MJ, Koes BW. The Effect of Tai Chi Chuan in Reducing Falls among Elderly People: Design of a Randomized Clinical Trial in the Netherlands [Isrctn98840266]. *BMC Geriatrics*. (2006) 6. doi:10.1186/1471-2318-6-6 |
| **Incomplete data(n=23)** | |
| 1 | Aviles J, Allin LJ, Alexander NB, Mullekom JV, Nussbaum MA, Madigan ML, et al. Comparison of Treadmill Trip-Like Training Versus Tai Chi to Improve Reactive Balance among Independent Older Adult Residents of Senior Housing: A Pilot Controlled Trial. *Journals of Gerontology Series A: Biological Sciences & Medical Sciences*. (2019) 74:1497-503. doi:10.1093/gerona/glz018 |
| 2 | González López-Arza MV, Varela-Donoso E, Montanero-Fernández J, Rodríguez-Mansilla J, González-Sánchez B, González López-Arza L. Qigong Improves Balance in Young Women: A Pilot Study. *Journal of Chinese Integrative Medicine*. (2013) 11:241-5. doi:10.3736/jintegrmed2013038 |
| 3 | Gow BJ, Hausdorff JM, Manor B, Lipsitz LA, Macklin EA, Bonato P, et al. Can Tai Chi Training Impact Fractal Stride Time Dynamics, an Index of Gait Health, in Older Adults? Cross-Sectional and Randomized Trial Studies. *PloS one*. (2017) 12:e0186212. doi:10.1371/journal.pone.0186212 |
| 4 | Holmes ML, Manor B, Hsieh W-h, Hu K, Lipsitz LA, Li L. Tai Chi Training Reduced Coupling between Respiration and Postural Control. *Neuroscience letters*. (2016) 610:60-5. doi:10.1016/j.neulet.2015.10.053 |
| 5 | Hu X, Lyu S, Mao M, Zhang J, Sun W, Zhang C, et al. Effects of Eight Methods and Five Steps of Tai Chi Practice on Balance Control among Older Adults. *Motor Control*. (2021) 25:616-30. doi:10.1123/mc.2021-0022 |
| 6 | Hwang HF, Chen SJ, Lee-Hsieh J, Chien DK, Chen CY, Lin MR. Effects of Home-Based Tai Chi and Lower Extremity Training and Self-Practice on Falls and Functional Outcomes in Older Fallers from the Emergency Department - a Randomized Controlled Trial. *Journal of the American Geriatrics Society*. (2016) 64:518-25. doi:10.1111/jgs.13952 |
| 7 | Irct20180426039425N. Effect of Taichi's Exercise on Physical Performance and Fall Rates. *https://trialsearch.who.int/Trial2.aspx?TrialID=IRCT20180426039425N1*. (2018). |
| 8 | Lelard T, Doutrellot PL, David P, Ahmaidi S. Effects of a 12-Week Tai Chi Chuan Program Versus a Balance Training Program on Postural Control and Walking Ability in Older People. *Archives of Physical Medicine and Rehabilitation*. (2010) 91:9-14. doi:10.1016/j.apmr.2009.09.014 |
| 9 | Li F, Harmer P, Eckstrom E, Fitzgerald K, Chou LS, Liu Y. Effectiveness of Tai Ji Quan Vs Multimodal and Stretching Exercise Interventions for Reducing Injurious Falls in Older Adults at High Risk of Falling: Follow-up Analysis of a Randomized Clinical Trial. *JAMA Network Open*. (2019) 2. doi:10.1001/jamanetworkopen.2018.8280 |
| 10 | Liye Z, Chaoyi W, Zuguo T, Huiru W, Yankai S. Effect of Yang-Style Tai Chi on Gait Parameters and Musculoskeletal Flexibility in Healthy Chinese Older Women. *Sports (2075-4663)*. (2017) 5:52. |
| 11 | Lu X, Siu KC, Fu SN, Hui-Chan CWY, Tsang WWN. Effects of Tai Chi Training on Postural Control and Cognitive Performance While Dual Tasking-a Randomized Clinical Trial. *Journal of Complementary and Integrative Medicine*. (2016) 13:181-7. doi:10.1515/jcim-2015-0084 |
| 12 | Mihay LM, Boggs KM, Breck AJ, Dokken EL, NaThalang GC. The Effect of Tai Chi Inspired Exercise Compared to Strength Training: A Pilot Study of Elderly Retired Community Dwellers. *Physical and Occupational Therapy in Geriatrics*. (2006) 24:13-26. doi:10.1300/J148v24n03_02 |
| 13 | Pakzad-Mayer Y, Chan YS, Jang JT, Mayer PK. Does Practicing Tai Chi Chuan Make a Difference for Postural Balance in the Elderly? A Pilot Study. *Hat das Praktizieren von Tai Chi Chuan einen Einfluss auf die posturale Balance bei Senioren? Eine Teststudie.* (2019) 70:159-64. |
| 14 | Solianik R, Brazaitis M, Čekanauskaitė-Krušnauskienė A. Tai Chi Effects on Balance in Older Adults: The Role of Sustained Attention and Myokines. *The Journal of sports medicine and physical fitness*. (2022) 62:1512-8. doi:10.23736/S0022-4707.21.12990-1 |
| 15 | Tsang WW, Hui-Chan CW. Effects of Tai Chi on Joint Proprioception and Stability Limits in Elderly Subjects. *Med Sci Sports Exerc*. (2003) 35:1962-71. doi:10.1249/01.Mss.0000099110.17311.A2 |
| 16 | Voukelatos A, Cumming RG, Lord SR, Rissel C. A Randomized, Controlled Trial of Tai Chi for the Prevention of Falls: The Central Sydney Tai Chi Trial. *Journal of the American Geriatrics Society*. (2007) 55:1185-91. doi:10.1111/j.1532-5415.2007.01244.x |
| 17 | Wayne PM, Gow BJ, Costa MD, Peng CK, Lipsitz LA, Hausdorff JM, et al. Complexity-Based Measures Inform Effects of Tai Chi Training on Standing Postural Control: Cross-Sectional and Randomized Trial Studies. *PLoS ONE*. (2014) 9. doi:10.1371/journal.pone.0114731 |
| 18 | Wayne PM, Gow BJ, Hou FZ, Ma Y, Hausdorff JM, Lo J, et al. Tai Chi Training's Effect on Lower Extremity Muscle Co-Contraction During Single- and Dual-Task Gait: Cross-Sectional and Randomized Trial Studies. *Plos One*. (2021) 16. doi:10.1371/journal.pone.0242963 |
| 19 | Wong TWL. Feasibility and Preliminary Efficacy of Ai Chi Aquatic Exercise Training in Hong Kong's Older Adults with Risk of Falling: Design and Methodology of a Randomized Controlled Trial. *Contemporary clinical trials communications*. (2019) 15. doi:10.1016/j.conctc.2019.100376 |
| 20 | Woo J, Hong A, Lau E, Lynn H. A Randomised Controlled Trial of Tai Chi and Resistance Exercise on Bone Health, Muscle Strength and Balance in Community-Living Elderly People. *Age and Ageing*. (2007) 36:262-8. doi:10.1093/ageing/afm005 |
| 21 | Yang Y, Verkuilen JV, Rosengren KS, Grubisich SA, Reed MR, Hsiao-Wecksler ET. Effect of Combined Taiji and Qigong Training on Balance Mechanisms: A Randomized Controlled Trial of Older Adults. *Medical Science Monitor*. (2007) 13:CR339-CR48. |
| 22 | Zeeuwe PEM, Verhagen AP, Bierma-Zeinstra SMA, Van Rossum E, Faber MJ, Koes BW. The Effect of Tai Chi Chuan in Reducing Falls among Elderly People: Design of a Randomized Clinical Trial in the Netherlands [Isrctn98840266]. *BMC Geriatrics*. (2006) 6. doi:10.1186/1471-2318-6-6 |
| 23 | Zou L, Wang C, Tian Z, Wang H, Shu Y. Effect of Yang-Style Tai Chi on Gait Parameters and Musculoskeletal Flexibility in Healthy Chinese Older Women. *Sports (Basel, Switzerland)*. (2017) 5. doi:10.3390/sports5030052 |
| **No data of interest(n=16)** | |
| 1 | Adcock M, Sonder F, Schättin A, Gennaro F, de Bruin ED. A Usability Study of a Multicomponent Video Game-Based Training for Older Adults. *European review of aging and physical activity : official journal of the European Group for Research into Elderly and Physical Activity*. (2020) 17:3. doi:10.1186/s11556-019-0233-2 |
| 2 | Benyi M, Vamos M, Meszaros L, Czudar P, Rakos-Zichy P. Traditional Physical Exercise and Tai Chi Comparative Study among Older People in Hungary. *Injury Prevention (1353-8047)*. (2010) 16:A148-A9. |
| 3 | Blake H, Hawley H. Effects of Tai Chi Exercise on Physical and Psychological Health of Older People. *Current Aging Science*. (2012) 5:19-27. doi:10.2174/1874609811205010019 |
| 4 | Chen Y, Qin J, Tao L, Liu Z, Huang J, Liu W, et al. Effects of Tai Chi Chuan on Cognitive Function in Adults 60 Years or Older with Type 2 Diabetes and Mild Cognitive Impairment in China: A Randomized Clinical Trial. *JAMA Network Open*. (2023) 6:E237004. doi:10.1001/jamanetworkopen.2023.7004 |
| 5 | Dawei L, Jiajie L. Effect of Tai-Ji Practice on the Health of the Elderly. *EFECTO DE LA PRÁCTICA DE TAI-JI EN LA SALUD DE LAS PERSONAS MAYORES.* (2023) 29:1-4. |
| 6 | Du Y, Roberts P, Liu W. Facilitators and Barriers of Tai Chi Practice in Community-Dwelling Older Adults: Qualitative Study. *Asian/Pacific Island nursing journal*. (2023) 7:e42195. doi:10.2196/42195 |
| 7 | Frye B, Scheinthal S, Kemarskaya T, Pruchno R. Tai Chi and Low Impact Exercise: Effects on the Physical Functioning and Psychological Well-Being of Older People. *Journal of Applied Gerontology*. (2007) 26:433-53. doi:10.1177/0733464807306915 |
| 8 | Gogulla S, Lemke N, Hauer K. Effects of Physical Activity and Physical Training on the Psychological Status of Older Persons with and without Cognitive Impairment. *Zeitschrift fur Gerontologie und Geriatrie*. (2012) 45:279-89. doi:10.1007/s00391-012-0347-x |
| 9 | Hasegawa M, Harada T, Muro M. Effect of Short Term Tonic Vibration in Agonist Muscle on Autonomic Nervous Modulation in Elderly Novice Tai Chi Practitioners. *Respiration and Circulation*. (2007) 55:1375-80. |
| 10 | Jianwei D, Kuan W, Tongbo C, Lejun W, Shengnian Z, Wenxin N. Tai Chi Is Safe and Effective for the Hip Joint: A Biomechanical Perspective. *Journal of Aging & Physical Activity*. (2020) 28:415-25. |
| 11 | Kressig RW, Beauchet O, Tharicharu J. T'ai Chi in the Elderly: Practical Aspects. *Revue médicale de la Suisse romande*. (2003) 123:671-5. |
| 12 | Lei G, Bailey R. Therapeutic Benefits of Tai Chi for Older Adults. *Palaestra*. (2022) 36:21-7. |
| 13 | Lough M, Manor B, Gagnon M, Cupples A, Wayne PM, Lipsitz LA. Functional Benefits of Tai Chi Training within Senior Housing Facilities. *Journal of the American Geriatrics Society*. (2014) 62:S99-S100. doi:10.1111/jgs.12870 |
| 14 | Mercuris K, Lowry K, Espey S. Does Instructor Experience Impact Balance and Health-Related Quality of Life in Healthy Older Adult Participants Following a Tai Chi for Arthritis Program? *Journal of community health nursing*. (2020) 37:26-34. doi:10.1080/07370016.2019.1693136 |
| 15 | Training TC, Ma Y, Hou F, Gow B, Hausdorff J, Lo J, et al. Reduced Muscle Cocontraction in Older Adults Following Long-and Short-Term. *Global Advances in Health and Medicine*. (2020) 9:24. doi:10.1177/2164956120912849 |
| 16 | Zechner MR, McDonald M, King-Rumelhart T, Jahnke R, Monroy-Miller C. Engaging Older Adults Using Tai Chi at a Psychiatric Hospital. *American Journal of Psychiatric Rehabilitation*. (2018) 21:344-54. |
| **Older < 60 age(n=7)** | |
| 1 | Oddsson LIE, Bisson T, Cohen HS, Iloputaife I, Jacobs L, Kung D, et al. Extended Effects of a Wearable Sensory Prosthesis on Gait, Balance Function and Falls after 26 Weeks of Use in Persons with Peripheral Neuropathy and High Fall Risk-the Walk2wellness Trial. *Frontiers in Aging Neuroscience*. (2022) 14. doi:10.3389/fnagi.2022.931048 |
| 2 | Pan S, Kairy D, Corriveau H, Tousignant M. Adapting Tai Chi for Upper Limb Rehabilitation Post Stroke: A Feasibility Study. *Medicines (Basel, Switzerland)*. (2017) 4. doi:10.3390/medicines4040072 |
| 3 | Rogers CE, Keller C, Larkey LK, Ainsworth BE. A Randomized Controlled Trial to Determine the Efficacy of Sign Chi Do Exercise on Adaptation to Aging. *Research in Gerontological Nursing*. (2012) 5:101-13. doi:10.3928/19404921-20110706-01 |
| 4 | Taylor-Piliae RE, Coull BM. Community-Based Yang-Style Tai Chi Is Safe and Feasible in Chronic Stroke: A Pilot Study. *Clinical rehabilitation*. (2012) 26:121-31. doi:10.1177/0269215511419381 |
| 5 | Uhlig T, Fongen C, Steen E, Christie A, Ødegård S. Exploring Tai Chi in Rheumatoid Arthritis: A Quantitative and Qualitative Study. *BMC musculoskeletal disorders*. (2010) 11:43. doi:10.1186/1471-2474-11-43 |
| 6 | Wang N, Zhang X, Xiang Y-B, Li H, Yang G, Gao J, et al. Associations of Tai Chi, Walking, and Jogging with Mortality in Chinese Men. *American journal of epidemiology*. (2013) 178:791-6. doi:10.1093/aje/kwt050 |
| 7 | Wood LRJ, Blagojevic-Bucknall M, Stynes S, D'Cruz D, Mullis R, Whittle R, et al. Impairment-Targeted Exercises for Older Adults with Knee Pain: A Proof-of-Principle Study (Target-Knee-Pain). *Bmc Musculoskeletal Disorders*. (2016) 17. doi:10.1186/s12891-016-0899-9 |
| **Review,etc (n = 13)** | |
| 1 | Binns E, Taylor D, Hale L, Schluter P, Waters D, McCracken H, et al. Tai Chi for Falls Prevention: The Effect of Group Exercise. *Physiotherapy (United Kingdom)*. (2011) 97:eS132. doi:10.1016/j.physio.2011.04.002 |
| 2 | Corriveau H, Tousignant M, Roy PM, Tremblay-Boudreault V, Desrosiers J, Dubuc N, et al. Efficacy of Supervised Tai Chi Exercises Compared to Physiotherapy Program in Fall Prevention for Frail Older Adults: A Randomised Trial. *Physiotherapy (United Kingdom)*. (2011) 97:eS239. doi:10.1016/j.physio.2011.04.002 |
| 3 | Duenas EP, Ramirez LP, Ponce E, Curcio CL. Effect on Fear of Falling and Functionality of Three Intervention Programs. A Randomised Clinical Trial. *Revista espanola de geriatria y gerontologia*. (2018) (no pagination). doi:10.1016/j.regg.2018.09.013 |
| 4 | Furlong D, Olmstead R, Irwin MR. Tai Chi Chih and Regulation of Sympathovagal Balance in Older Adults: A Randomized Controlled Trial. *Psychosomatic Medicine*. (2014) 76:A-2. doi:10.1097/PSY.0000000000000057 |
| 5 | Harmer P, Li F. Tai Chi and Falls Prevention in Older People. 2008, 52((Harmer P., pharmer@willamette.edu) Exercise Science, Sports Medicine, Willamette University, Salem, OR, United States): 124-34 |
| 6 | Godziejewska-Zawada M. Obesity and Diabetes in Menopause: Prevention and Therapeutic Approach. *Menopause Review-Przeglad Menopauzalny*. (2013) 12:5-9. doi:10.5114/pm.2013.33413 |
| 7 | Gravesande J, de Oliveira LA, Malik N, Vrkljan B, Zheng R, Gardner PM, et al. Feasibility, Usability, and Acceptability of Online Mind-Body Exercise Programs for Older Adults: A Scoping Review. *Journal of Integrative and Complementary Medicine*. (2023) 29:538-49. doi:10.1089/jicm.2022.0822 |
| 8 | Hasan F, Tu Y-K, Lin C-M, Chuang L-P, Jeng C, Yuliana LT, et al. Comparative Efficacy of Exercise Regimens on Sleep Quality in Older Adults: A Systematic Review and Network Meta-Analysis. *Sleep medicine reviews*. (2022) 65:101673. doi:10.1016/j.smrv.2022.101673 |
| 9 | Lough M, Manor B, Gagnon M, Iloputaife I, Wayne P, Lipsitz L. Tai Chi Training Increases the Complexity of Standing Postural Control in Frail Older Adults. *Journal of the American Geriatrics Society*. (2012) 60:S102-S3. doi:10.1111/j.1532-5415.2012.04000.x |
| 10 | Teixeira R, Pérez L, Lambeck J, Neto F. The Influence of Ai Chi on Balance and Fear of Falling in Older Adults: A Randomized Clinical Trial. *Physiotherapy (United Kingdom)*. (2011) 97:eS654. doi:10.1016/j.physio.2011.04.002 |
| 11 | Vilpunaho T, Kroger H, Honkanen R, Koivumaa-Honkanen H, Sirola J, Kuvaja-Kollner V, et al. Randomised Controlled Trial (Rct) Study Design for a Large-Scale Municipal Fall Prevention Exercise Programme in Community-Living Older Women: Study Protocol for the Kuopio Fall Prevention Study (Kfps). *BMJ open*. (2019) 9:e028716. doi:10.1136/bmjopen-2018-028716 |
| 12 | Zhou Z, Zhou R, Li K, Zhu Y, Zhang Z, Luo Y, et al. Effects of Tai Chi on Physiology, Balance and Quality of Life in Patients with Type 2 Diabetes: A Systematic Review and Meta-Analysis. *Journal of rehabilitation medicine*. (2019) 51:405-17. doi:10.2340/16501977-2555 |
| 13 | Zwick D, Rochelle A, Choksi A, Domowicz J. Evaluation and Treatment of Balance in the Elderly: A Review of the Efficacy of the Berg Balance Test and Tai Chi Quan. *NeuroRehabilitation*. (2000) 15:49-56. doi:10.3233/nre-2000-15103 |
| **Conference papers, other reports, etc(n = 5)** | |
| 1 | Lough M, Manor B, Gagnon M, Iloputaife I, Wayne P, Lipsitz L. Tai Chi Training Increases the Complexity of Standing Postural Control in Frail Older Adults. *Journal of the American Geriatrics Society*. (2012) 60:S102-S3. doi:10.1111/j.1532-5415.2012.04000.x |
| 2 | Teixeira R, Pérez L, Lambeck J, Neto F. The Influence of Ai Chi on Balance and Fear of Falling in Older Adults: A Randomized Clinical Trial. *Physiotherapy (United Kingdom)*. (2011) 97:eS654. doi:10.1016/j.physio.2011.04.002 |
| 3 | Corriveau H, Tousignant M, Roy PM, Tremblay-Boudreault V, Desrosiers J, Dubuc N, et al. Efficacy of Supervised Tai Chi Exercises Compared to Physiotherapy Program in Fall Prevention for Frail Older Adults: A Randomised Trial. *Physiotherapy (United Kingdom)*. (2011) 97:eS239. doi:10.1016/j.physio.2011.04.002 |
| 4 | Binns E, Taylor D, Hale L, Schluter P, Waters D, McCracken H, et al. Tai Chi for Falls Prevention: The Effect of Group Exercise. *Physiotherapy (united kingdom)*. (2011) 97:eS132. doi:10.1016/j.physio.2011.04.002 |
| 5 | Furlong D, Olmstead R, Irwin MR. Tai Chi Chih and Regulation of Sympathovagal Balance in Older Adults: A Randomized Controlled Trial. *Psychosomatic Medicine*. (2014) 76:A-2. doi:10.1097/PSY.0000000000000057 |

**Table S8 Recommendations for future studies on the effect of Tai Chi on balance performance**

| **Study**  **Characteristic** | **Recommendations** |
| --- | --- |
| **Population** | Research in different cultural backgrounds:  Tai Chi originated in China, and its philosophy and practice may differ from Western cultures. Future research could consider assessing the acceptance and effectiveness of Tai Chi in different cultural backgrounds, for example, through cross-cultural comparative studies to analyse the effects of taijiquan on balance among older adults in different cultures. This may require adapting teaching methods, environments and study designs to suit the needs and preferences of different cultures. |
| **Outcomes** | Increase research on reactive balance:  Reactive balance refers to an individual's ability to cope with unexpected perturbations critical to overall balance performance. Current research has focused on static and dynamic balance, while more research needs to be conducted on reactive balance. Future studies should assess the effect of Tai Chi on reactive balance by designing specific experimental conditions, such as CoP displacement after an unexpected perturbation. |
| **Study design** | Quantitative analysis of publication bias:  Publication bias can lead to inaccurate results in meta-analyses, as studies with positive results are more likely to be published. In meta-analyses, assessing publication bias through relevant statistical tests (e.g., Egger's test) is necessary. Future studies should use these statistical methods to quantify publication bias and assess the robustness of the results. |
| **Other recommendations** | Repeatedly experimenting with the same participants, despite the associated increase in cost and methodological complexity, bolsters the results' reliability. Finally, given the predominant focus of the present study on China, it is advisable to conduct further research on this subject in diverse geographical settings across various countries to enhance the robustness and applicability of the results. |


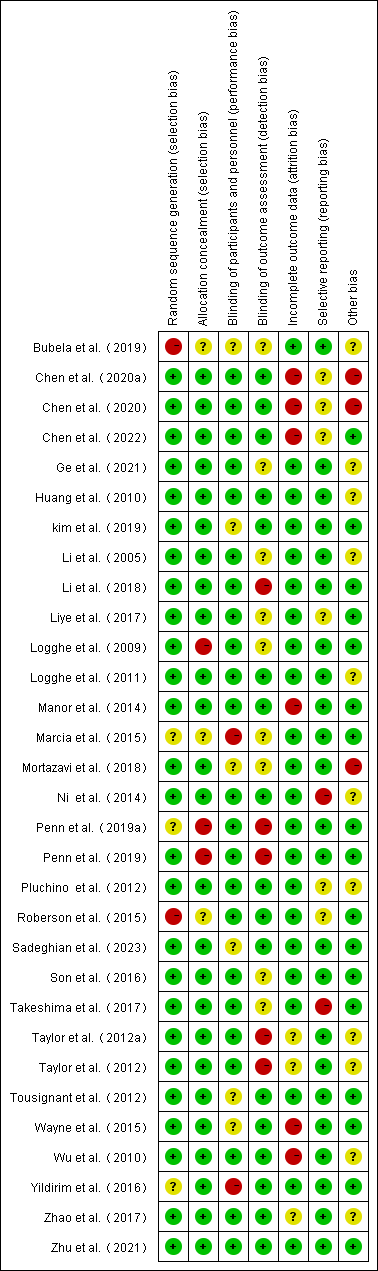


**Figure S1 Risk of bias assessment.**

**Table S9. PRISMA Abstracts checklist**

| **Section and Topic** | **Item #** | **Checklist item** | **Reported (Yes/No)** |
| --- | --- | --- | --- |
| **TITLE** | | |  |
| Title | 1 | Identify the report as a systematic review. | Yes |
| **BACKGROUND** | | |  |
| Objectives | 2 | Provide an explicit statement of the main objective(s) or question(s) the review addresses. | Yes |
| **METHODS** | | |  |
| Eligibility criteria | 3 | Specify the inclusion and exclusion criteria for the review. | Yes |
| Information sources | 4 | Specify the information sources (e.g. databases, registers) used to identify studies and the date when each was last searched. | Yes |
| Risk of bias | 5 | Specify the methods used to assess risk of bias in the included studies. | No |
| Synthesis of results | 6 | Specify the methods used to present and synthesise results. | Yes |
| **RESULTS** | | |  |
| Included studies | 7 | Give the total number of included studies and participants and summarise relevant characteristics of studies. | Yes |
| Synthesis of results | 8 | Present results for main outcomes, preferably indicating the number of included studies and participants for each. If meta-analysis was done, report the summary estimate and confidence/credible interval. If comparing groups, indicate the direction of the effect (i.e. which group is favoured). | Yes |
| **DISCUSSION** | | |  |
| Limitations of evidence | 9 | Provide a brief summary of the limitations of the evidence included in the review (e.g. study risk of bias, inconsistency and imprecision). | No |
| Interpretation | 10 | Provide a general interpretation of the results and important implications. | Yes |
| **OTHER** | | |  |
| Funding | 11 | Specify the primary source of funding for the review. | No |
| Registration | 12 | Provide the register name and registration number. | Yes |

**Table S10. PRISMA checklist.**

| **Section and Topic** | **Item #** | **Checklist item** | **Location where item is reported** |
| --- | --- | --- | --- |
| **TITLE** | | |  |
| Title | 1 | Identify the report as a systematic review. | P.1 |
| **ABSTRACT** | | |  |
| Abstract | 2 | See the PRISMA 2020 for Abstracts checklist. | P.1 |
| **INTRODUCTION** | | |  |
| Rationale | 3 | Describe the rationale for the review in the context of existing knowledge. | P.2 |
| Objectives | 4 | Provide an explicit statement of the objective(s) or question(s) the review addresses. | P.2 |
| **METHODS** | | |  |
| Eligibility criteria | 5 | Specify the inclusion and exclusion criteria for the review and how studies were grouped for the syntheses. | P.2-3 |
| Information sources | 6 | Specify all databases, registers, websites, organisations, reference lists and other sources searched or consulted to identify studies. Specify the date when each source was last searched or consulted. | P.2 |
| Search strategy | 7 | Present the full search strategies for all databases, registers and websites, including any filters and limits used. | Table S1-S6  (Supplementary material) |
| Selection process | 8 | Specify the methods used to decide whether a study met the inclusion criteria of the review, including how many reviewers screened each record and each report retrieved, whether they worked independently, and if applicable, details of automation tools used in the process. | P.3 |
| Data collection process | 9 | Specify the methods used to collect data from reports, including how many reviewers collected data from each report, whether they worked independently, any processes for obtaining or confirming data from study investigators, and if applicable, details of automation tools used in the process. | P.3 |
| Data items | 10a | List and define all outcomes for which data were sought. Specify whether all results that were compatible with each outcome domain in each study were sought (e.g. for all measures, time points, analyses), and if not, the methods used to decide which results to collect. | P.2-3 |
|  | 10b | List and define all other variables for which data were sought (e.g. participant and intervention characteristics, funding sources). Describe any assumptions made about any missing or unclear information. | P.2-3 |
| Study risk of bias assessment | 11 | Specify the methods used to assess risk of bias in the included studies, including details of the tool(s) used, how many reviewers assessed each study and whether they worked independently, and if applicable, details of automation tools used in the process. | P.3 |
| Effect measures | 12 | Specify for each outcome the effect measure(s) (e.g. risk ratio, mean difference) used in the synthesis or presentation of results. | P.2-3 |
| Synthesis methods | 13a | Describe the processes used to decide which studies were eligible for each synthesis (e.g. tabulating the study intervention characteristics and comparing against the planned groups for each synthesis (item #5)). | P.3 |
|  | 13b | Describe any methods required to prepare the data for presentation or synthesis, such as handling of missing summary statistics, or data conversions. | P.3 |
|  | 13c | Describe any methods used to tabulate or visually display results of individual studies and syntheses. | P.3 |
|  | 13d | Describe any methods used to synthesize results and provide a rationale for the choice(s). If meta-analysis was performed, describe the model(s), method(s) to identify the presence and extent of statistical heterogeneity, and software package(s) used. | P.3 |
|  | 13e | Describe any methods used to explore possible causes of heterogeneity among study results (e.g. subgroup analysis, meta-regression). | P.3 |
|  | 13f | Describe any sensitivity analyses conducted to assess robustness of the synthesized results. | P.3 |
| Reporting bias assessment | 14 | Describe any methods used to assess risk of bias due to missing results in a synthesis (arising from reporting biases). | P.3 |
| Certainty assessment | 15 | Describe any methods used to assess certainty (or confidence) in the body of evidence for an outcome. | P.3 |
| **RESULTS** | | |  |
| Study selection | 16a | Describe the results of the search and selection process, from the number of records identified in the search to the number of studies included in the review, ideally using a flow diagram. | P.3-4 |
|  | 16b | Cite studies that might appear to meet the inclusion criteria, but which were excluded, and explain why they were excluded. | Table S7 (Supplementary material) |
| Study characteristics | 17 | Cite each included study and present its characteristics. | P.3-5 |
| Risk of bias in studies | 18 | Present assessments of risk of bias for each included study. | P.5-6 |
| Results of individual studies | 19 | For all outcomes, present, for each study: (a) summary statistics for each group (where appropriate) and (b) an effect estimate and its precision (e.g. confidence/credible interval), ideally using structured tables or plots. | P.5-6 |
| Results of syntheses | 20a | For each synthesis, briefly summarize the characteristics and risk of bias among contributing studies. | P.5-6 |
|  | 20b | Present results of all statistical syntheses conducted. If meta-analysis was done, present for each the summary estimate and its precision (e.g. confidence/credible interval) and measures of statistical heterogeneity. If comparing groups, describe the direction of the effect. | P.5-6 |
|  | 20c | Present results of all investigations of possible causes of heterogeneity among study results. | P.5-6 |
|  | 20d | Present results of all sensitivity analyses conducted to assess the robustness of the synthesized results. | P.4-6 |
| Reporting biases | 21 | Present assessments of risk of bias due to missing results (arising from reporting biases) for each synthesis assessed. | P.4-6 |
| Certainty of evidence | 22 | Present assessments of certainty (or confidence) in the body of evidence for each outcome assessed. | P.5-6 |
| **DISCUSSION** | | |  |
| Discussion | 23a | Provide a general interpretation of the results in the context of other evidence. | P.6-10 |
|  | 23b | Discuss any limitations of the evidence included in the review. | P.6-10 |
|  | 23c | Discuss any limitations of the review processes used. | P.10-11 |
|  | 23d | Discuss implications of the results for practice, policy, and future research. | P.10-11 |
| **OTHER INFORMATION** | | |  |
| Registration and protocol | 24a | Provide registration information for the review, including register name and registration number, or state that the review was not registered. | P.1 |
|  | 24b | Indicate where the review protocol can be accessed, or state that a protocol was not prepared. | P.1 |
|  | 24c | Describe and explain any amendments to information provided at registration or in the protocol. | Not applicable |
| Support | 25 | Describe sources of financial or non-financial support for the review, and the role of the funders or sponsors in the review. | Not applicable |
| Competing interests | 26 | Declare any competing interests of review authors. | P.11-12 |
| Availability of data, code and other materials | 27 | Report which of the following are publicly available and where they can be found: template data collection forms; data extracted from included studies; data used for all analyses; analytic code; any other materials used in the review. | P.12 |
